# Why Does Space Feel the Way it Does? Towards a Principled Account of Spatial Experience

**DOI:** 10.3390/e21121160

**Published:** 2019-11-27

**Authors:** Andrew Haun, Giulio Tononi

**Affiliations:** Department of Psychiatry, University of Wisconsin-Madison, Madison, WI 53719, USA

**Keywords:** integrated information theory, consciousness, qualia, phenomenology, causal structure, grid networks

## Abstract

There must be a reason why an experience feels the way it does. A good place to begin addressing this question is spatial experience, because it may be more penetrable by introspection than other qualities of consciousness such as color or pain. Moreover, much of experience is spatial, from that of our body to the visual world, which appears as if painted on an extended canvas in front of our eyes. Because it is ‘right there’, we usually take space for granted and overlook its qualitative properties. However, we should realize that a great number of phenomenal distinctions and relations are required for the canvas of space to feel ‘extended’. Here we argue that, to be experienced as extended, the canvas of space must be composed of countless spots, here and there, small and large, and these spots must be related to each other in a characteristic manner through connection, fusion, and inclusion. Other aspects of the structure of spatial experience follow from extendedness: every spot can be experienced as enclosing a particular region, with its particular location, size, boundary, and distance from other spots. We then propose an account of the phenomenal properties of spatial experiences based on integrated information theory (IIT). The theory provides a principled approach for characterizing both the quantity and quality of experience by unfolding the cause-effect structure of a physical substrate. Specifically, we show that a simple simulated substrate of units connected in a grid-like manner yields a cause-effect structure whose properties can account for the main properties of spatial experience. These results uphold the hypothesis that our experience of space is supported by brain areas whose units are linked by a grid-like connectivity. They also predict that changes in connectivity, even in the absence of changes in activity, should lead to a warping of experienced space. To the extent that this approach provides an initial account of phenomenal space, it may also serve as a starting point for investigating other aspects of the quality of experience and their physical correspondents.

## 1. Introduction

**Figure 1:**
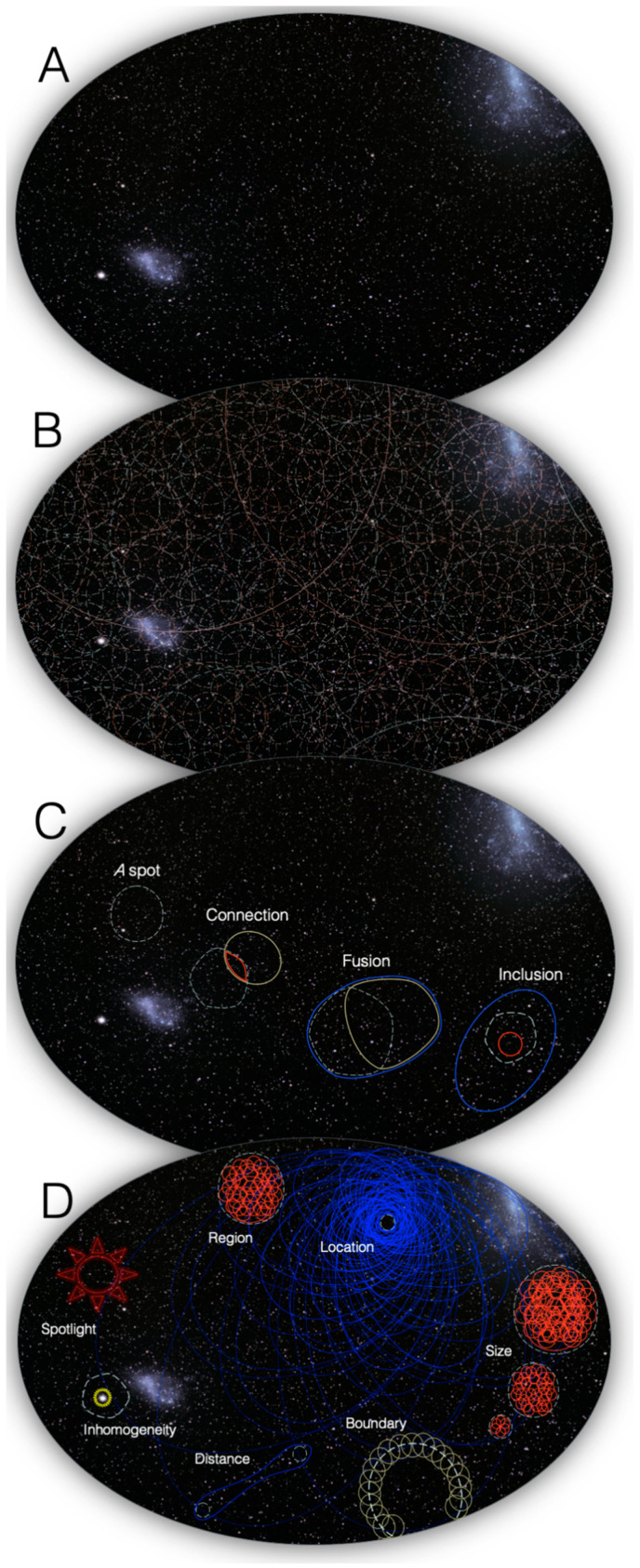
Phenomenology of spatial experience. (**A**) The extended canvas of the night sky. (**B**) *Spots*. Within the canvas, one can distinguish ‘spots’ (dashed outlines) anywhere and of any size (indicated by different colors of the dashing). (**C**) Fundamental properties of the phenomenal structure of spatial experience. Every spot overlaps itself and relates to other spots in a way that is characteristically *extended*: (a) they are *connected* with other partially overlapping spots (light brown) so that their intersection is also a spot (red); (b) they are *fused* with other partially overlapping spots (light brown) so that their union is also a spot (blue). (c) they *include* other spots, both *up* (blue) and *down* (red); (**D**) Sub-structures of spatial experience. Clock-wise from the top: a spot’s *region* is given by the spots it includes; its *location* by the spots that include it; its *size* by the number of spots in its region; its *boundary* by the set of smallest spots that connect to it; the *distance* between two spots by the smallest spot that extends to both; spots can encompass a *inhomogeneity* in the structure of space and they can be *highlighted* by the spotlight of attention. (photo credit, ESO/S.Brunier).

What can account for the quality of experience? Take a glimpse at the night sky (Figure 1): why does the experience feel subjectively the way it does—like an extended canvas sprinkled with tiny spots at many distinct locations? Then look at the sky on a bright day: why does its vivid blue color feel the way it does? Or why does pain feel painful? And why does a face look like a face? Eventually, a scientific understanding of consciousness should provide a principled, testable answer to such questions, but it is not easy to see how one could even begin to approach them. Indeed, the subjective, qualitative feeling of color or pain—“what it is like” to experience the blueness of the sky or the painfulness of pain [1]—is regularly invoked to highlight the seemingly unbridgeable explanatory gap between subjective “qualia” and the underlying neural mechanisms [2]. 

The paradigmatic examples used to illustrate the “hard problem” of consciousness are typically the subjective feeling of color or pain [3], rather than the subjective feeling of extendedness of the sky or of our own body. However, the feeling of extendedness is just as qualitative and in need of explanation as the feeling of color or pain [4,5]. It is also pervasive: much of our conscious life is spent anchored in body space, upon which we locate touch or pain, and immersed in visual space, which provides an extended canvas over which all the colors, the contours, and the objects we experience are painted, image after image (see Note 1 in Appendix B). Most important, spatial phenomenology may be a better place than color to attempt an initial account of how consciousness feels. For example, the experience of blue seems to be monolithically impenetrable to introspection, because it just seems inherently blue, with no obvious sign of an internal structure. By contrast, the experience of space is penetrable by introspection in a way that reveals a characteristic internal structure. As we will now argue, the canvas of space can be decomposed in terms of the phenomenal distinctions and relations that compose the experience and make it what it is.

Consider again looking at the night sky: the experience can be described roughly as a two-dimensional canvas (Figure 1A). Within the experiential canvas, one can readily *distinguish* ‘spots’ anywhere, of any size, as large as the entire firmament or as small as a single star (Figure 1B). Some reflection suggests that what endows the canvas with the characteristically spatial feeling of *extendedness* are certain properties of the *relations* among spots (Figure 1C): every spot overlaps with itself; for any spot, we can always find a spot that partially overlaps it such that their intersection is also a spot (connection); a spot that partially overlaps it such that their union is also a spot (fusion); and a spot that includes it or is included by it (inclusion). Further reflection suggests that other properties of the structure of spatial experience follow from the fundamental properties of connection, fusion, and inclusion (Figure 1D). Thus, a spot picks out a unique region of space, has a unique location within the overall space, a particular size, boundary, and distance from any other spot. Intriguingly, the canvas itself is not experienced as having a boundary: it extends ‘as far as one can see’, in the sense that there is no canvas exterior to it. Typically, the experience of space is embossed by local inhomogeneities, such as stars in the sky or blots on a canvas; moreover, particular regions of space can be highlighted by the spotlight of attention. However, it is important to realize that the spots and characteristic relations that compose phenomenal space are present whether or not some regions stand out: if a bright blot appears somewhere within the canvas, it cannot appear ‘out of nowhere’—a corresponding spot of the same size, with the appropriate relations to other spots, must already have been ‘there’, except that it was dark; similarly, the spot will remain ‘there’ even if the bright blot turns dark like the rest of the canvas (see Note 2 in Appendix B). Indeed, the experience of space does not depend on inhomogeneities within the canvas, such as bright stars or printed characters, but occurs even if the canvas is fully homogeneous, like a dark sky or an empty screen. Moreover, experiencing space does not require introspection or attention, but occurs without effort, as when walking on a country road with nothing particular in mind.

This crude phenomenal characterization of spatial experience is obviously incomplete—for example, the depth of visual space is not considered. It is also inevitably contaminated by non-spatial features—for example, we cannot help experiencing the color of the canvas, or avoid seeing bright spots as stars. However, we can still attempt to isolate spatial properties by abstracting over other features of experience. For example, within visual space, the canvas could be white or black, red or green, the inhomogeneities dark over a light background or the other way around, yet its phenomenal spatial properties remain largely the same, regardless of its other qualities. Spatial properties do not even need to be tied to the visual modality: extendedness applies just as well to body space, regardless of its local qualities such as touch, pressure, and so on. Thus, despite several limitations, it seems possible to capture the fundamental aspects of the experience of space. 

Can the structure of spatial experience be accounted for scientifically, in physical terms, bridging the explanatory gap in a way that is both principled and testable? More precisely, can phenomenal properties be accounted for in a way that connects them to specific substrates in the brain, which are known to determine the specific contents of consciousness? 

This paper presents an initial attempt at doing so by employing the principles of integrated information theory (IIT, [6,7]), which provides a comprehensive approach to characterize what consciousness is, what is required of a physical substrate to support it, and what determines the quality of particular experiences, including spatial ones.

Briefly, integrated information theory starts from the existence of one’s own consciousness, which is immediate and indubitable. The theory then identifies five essential properties that are also immediate, indubitable, and true of every conceivable experience, namely intrinsicality, composition, information, integration, and exclusion. Intrinsicality means that every experience is subjective—it is for the subject of experience, from its own intrinsic perspective, rather than for something extrinsic to it. Thus, the canvas of my phenomenal space is experienced by me, not by somebody else. Composition means that every experience is structured, being composed of phenomenal distinctions and relations. In the case of spatial experiences, distinctions can be taken as spots and their relations as the way spots connect, fuse, and include each other. Information means that every experience is the particular way it is. For example, the canvas of space may be homogeneous, or embossed by inhomogeneities such as stars, tree branches and leaves, or characters on the screen, in countless possible configurations. Integration means that every experience is unified, being irreducible to independent components. Thus, the canvas of space cannot be reduced to a left side and a right side that are experienced independently—if it were so, there would be two independent consciousnesses rather than one. Finally, exclusion means that every experience is definite—it contains what it contains, neither less nor more. In the case of space, the canvas contains all the spots and relations it contains—not just the left or the right side, or just a single spot in the middle or in the periphery—but it does not contain any spots beyond its borders, such as spots behind one’s head. Importantly, these five essential properties are true of every conceivable experience, not just of spatial ones. 

The theory then proceeds to characterize the requirements that must be satisfied by the physical substrate of our own consciousness. A physical substrate is intended as a system of connected units in a state, such as a set of active and inactive neurons in our brain. The cause-effect powers of a substrate are assessed by observations and manipulations: for example, neurons can be turned on or off by their inputs, and can turn on or off other neurons through their outputs. If every experience has the essential properties of being intrinsic, structured, specific, unified, and definite, its physical substrate must satisfy these properties in causal terms. Thus, intrinsicality means that a candidate substrate of consciousness, such as a set of neurons in the cerebral cortex, must have cause-effect power upon itself, rather than just with respect to sensory inputs and motor outputs. Composition means that one must consider the structure of intrinsic cause-effect power—how various combinations of neurons can have causes and effects within the system (causal distinctions) and how these distinctions overlap causally (causal relations). Information means that the causes and effects specified by various combinations of neurons are specific states of specific subsets of neurons, yielding a specific cause-effect structure. Integration means that causal distinctions and relations, as well as the overall cause-effect structure they compose, only exist if they are irreducible—if they cannot be reduced to independent causes and effects. Finally, exclusion means that causal distinctions and relations, as well as the cause-effect structure they compose, must be definite, containing what they contain—neither less nor more. What defines the set of neurons that constitute the physical substrate of consciousness—as opposed to any of its subsets or supersets—is being maximally irreducible, as measured by integrated information Φ.

On this basis, integrated information theory proposes an explanatory identity between a particular experience and the particular cause-effect structure specified by a physical substrate in its current state [6,7]. Applied to the experience of space, the proposed identity means the following: all the phenomenal distinctions and relations that compose the structure of phenomenal space must correspond one-to-one to causal distinctions and relations that compose the cause-effect structure specified by the neural substrate of spatial consciousness. In other words, all the spots that connect to each other, fuse with each other, and include each other to compose an extended structure characterized by regions, locations, sizes, boundaries, and distances, must have a correspondent in the cause-effect structure specified by the substrate of spatial experience in the brain. We emphasize that the goal is to account in physical terms for spatial experience as such—the causal distinctions and relations that compose it—rather than merely for how the brain represents external space and performs spatial functions. We further emphasize that the proposed correspondence is with the cause-effect structure unfolded from a neural substrate, not with the substrate as such.

A main reason for choosing space to assess the proposed identity between the properties of experience and those of cause-effect structures is that we can propose a plausible neural substrate, namely cortical areas that are organized in a grid-like manner, which are known to map both visual and body space [8]. As will be shown in the Results section, when properly unfolded to make all their causal powers explicit, grids specify cause-effect structures of extraordinary richness. This richness is needed to fully account for ‘what it is like’ to experience space—all the spots that are co-present in the mind, ordered by characteristic relations of connection, fusion, and inclusion to compose the feeling of an extended canvas.

In what follows, we apply the tools of integrated information theory to unfold the causal powers of simple grid-like substrates by characterizing the causal distinctions and relations they specify. While we examine primarily the cause-effect structures specified by small 1D grids due to their computational advantages, the findings can be generalized to larger, 2D grids. The results support the conjecture that the cause-effect structures specified by grid-like substrates, such as the 2D grids found in posterior cortical regions, could in principle account for the spatial aspects of experience.

## 2. Materials and Methods 

This section explains how, starting from a simple causal model, here a set of binary units connected as a 1D grid, one can unfold its cause-effect structure, which characterizes in full its cause-effect power. We first describe the causal model of the physical substrate. Next, we unfold the causal distinctions that represent the causes and effects specified by subsets of units. We then unfold the causal relations that represent overlaps among distinctions. By doing so systematically, we are in a position to characterize in full the cause-effect structure specified by the substrate in a given state. The general calculus of integrated information theory is laid out in previous work [6]. The present work adds several developments, primarily with respect to causal relations and the distance measure used to calculate irreducibility. Simulations were accomplished by using the freely available PyPhi software [9].

### 2.1. Causal Model of the Physical Substrate: A 1D Grid of Binary Units

**Figure 2:**
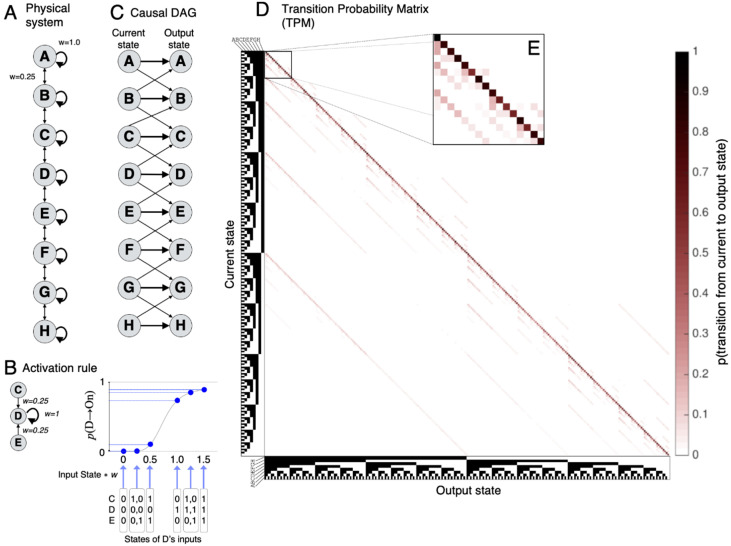
A grid, its units and states. (**A**) An 8-unit 1D grid network. Each unit is strongly connected to itself and weakly connected to its immediate neighbors, except for the two end units. (**B**) The activation rule for units in the grid. The current state of the inputs (1 for ‘On’ and 0 for ‘Off’) is weighted and summed by each unit to produce a probability of the unit’s output being On. Except for the end units, each grid unit has six distinct input levels producing different activation probabilities (blue dots), following a Naka-Rushton function of the input strength (continuous line). (**C**) The causal directed acyclic graph (DAG) of the grid. (**D**) Transition probability matrix (TPM) showing the probability with which the grid transitions from each possible current state (y-axis) to each possible output state (x-axis). (**E**) A close-up of the TPM shows that while the network prefers to stay in its current state, it can also drift into other states.

The causal model employed in this paper is a grid network, in the graph-theoretical sense of a lattice graph. The model is a 1D ‘line’ grid—in which each of 8 binary-state units is connected to itself and its two neighbors, except for units 1 and 8 at the border (Figure 2A). A line grid can be considered on its own or as a component of a larger 2D grid—for simplicity in this paper we focus on the cause-effect structure of the 1D grid alone. The unit’s activation function is a Naka-Rushton sigmoid [10]. For the *i*th unit in the grid, at every update the probability of being in state ‘On’ is (Figure 2B):

$$p\left(S_{i,t+1}\to ON\right)\;=\;\frac{{\left(\sum_{j}w_{i,j}S_{j,t}\right)}^{n}}{z+{\left(\sum_{j}w_{i,j}S_{j,t}\right)}^{n}}\;,\;\;p\left(S_{i,t+1}\to OFF\right)\;=\;1-p\left(S_{i,t+1}\to ON\right)$$

where the index *j* is over the inputs, *t* is the current state (and *t* + 1 the output state) and *w* is the connection strength, set to *w* = 1 for all self-connections and to ¼ for lateral connections.

The threshold parameter *z* was set to ¼. The exponent *n* was set to 5. Changing grid parameters (connection strength and degree as well as the activation rule) did not change the results qualitatively, although the current parameters were selected to obtain a cause-effect structure that was especially clear and easy to describe. The system’s input-output behavior, which can be represented as a causal directed acyclic graph (Figure 2C), is described completely by its transition probability matrix (TPM, Figure 2D). The strong self-connections make the system very stable, so that for any state the grid transitions most likely to the same state. However, because the Off state is more likely than the on state, over a long run the system will tend to converge towards states with patches of contiguous Off units.

According to integrated information theory, to correspond to an experience, a system in a state must specify a maximally irreducible, specific, compositional, intrinsic *cause-effect structure*, which is composed of *distinctions* (see Note 3 in Appendix B) and their *relations*. Both distinctions and relations, in turn, must be causal (make a difference) and satisfy intrinsicality (within the system), information (specificity), integration (irreducibility) and exclusion (maximal irreducibility).

### 2.2. Distinctions: Maximally Irreducible Cause-Effects

**Figure 3:**
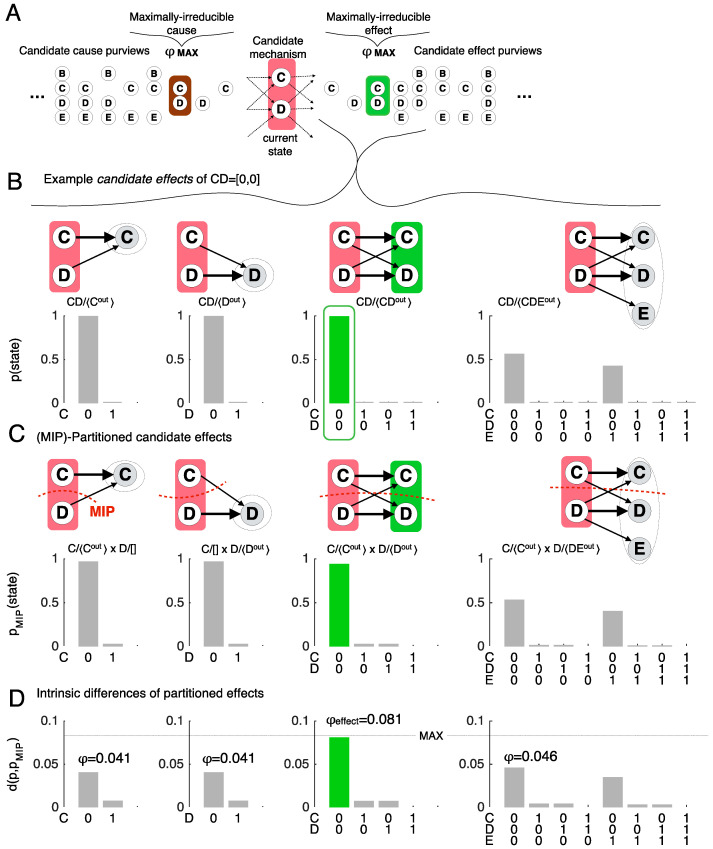
Characterization of a causal distinction. (**A**) Any subset of units can be considered as a candidate *mechanism*. One such candidate mechanism, illustrated here (in pink), is constituted by units CD in the current state CD = [0, 0] (0 in white and 1 in yellow). Any subset of units in a state can be considered as a candidate cause (in brown) or effect (in green). (**B**) Four candidate effect purviews. Each *effect* is represented as the probability *p* of a future state of a subset of units (a *purview*) given the current state of the mechanism, for example for CD^out^ = [0, 0], *p* = 0.996. (**C**) For each effect purview, the mechanism is partitioned (red dashed line) in the way that minimizes the difference between unpartitioned and partitioned effects. Each partitioned effect is represented as *p_MIP_*. (**D**) The *intrinsic difference d* between the partitioned and unpartitioned effects for the four candidate effects. The maximally-irreducible effect (the effect for which *d* is maximal) is CD^out^ = [0, 0].

To unfold the intrinsic cause-effect structure specified by the system, each subset of units is taken as a *candidate mechanism* and its outputs and inputs are identified to evaluate its cause-effect within the system. First consider the *effect* of a mechanism. Every subset of the outputs of the mechanism is taken as a candidate *purview* and its possible states as candidate *effects*. Each candidate effect is then evaluated for irreducibility, yielding the maximally irreducible effect. Algorithmically, this is done by first identifying the maximally irreducible state for any given purview and then comparing across all possible purviews. As an example, Figure 3 shows candidate mechanism CD, with the grid set into the all-Off state (A, B, C, … = [0, 0, 0, …]) (Figure 3A), and a small subset of candidate effects within the system, over purviews {C, D, CD, and CDE} (Figure 3B–D). Figure 3B shows how mechanism CD causally constrains the *effect repertoire* for each candidate purview. An effect repertoire is a probability distribution over the possible effects on the purview given the current state of the mechanism (indicated by the superscript ^c^), and is computed from the TPM [6]. 

*Irreducibility* is evaluated by cutting the mechanism and its purview apart [11] and assessing what difference the cut makes (Figure 3C). In the example of effect purview C^out^ (^out^ stands for output state) there are only three ways to cut the mechanism and its purview (each can be represented as a partition fraction, with mechanism units in the numerator and purview units in the denominator; the partition is indicated by the multiplication symbol, reflecting the independence of the parts):

cut the connection between *C^c^* and *C^out^* $(\frac{C^{c}}{\left[\;\right]}\times \frac{D^{c}}{C^{out}})$;
cut the connection between *D^c^* and *C^out^* $(\frac{C^{c}}{C^{out}}\times \frac{D^{c}}{\left[\;\right]})$;
cut both connections, $(\frac{C^{c}}{\left[\;\right]}\times \frac{D^{c}}{\left[\;\right]}\times \frac{\left[\;\right]}{C^{out}})$.

For each cut, a partitioned effect repertoire (Figure 3C) is computed by ‘noising’ the cut connections to obtain causal independence across the partition. Mathematically, this is done by causally marginalizing the constraints exerted by the mechanism across the cut [6]. The difference the cut makes from the perspective of the mechanism, between the repertoire *p* and the partitioned repertoire *p_cut_*, is assessed by the *intrinsic difference d* (Barbosa et al., in preparation; see Note 3):

(1) $$d\left(p,p_{cut}\right)\;=\;\underset{S}{\max}\left|p\left(S\right)\log\frac{p\left(S\right)}{p{\left(S\right)}_{cut}}\right|$$

To yield *intrinsic information*, the difference made by the mechanism must be a *specific effect*. For a given partition, this is the state *S* for which the difference is maximal (Figure 3D).

Since a mechanism cannot exist more than its ‘weakest link’, the irreducibility of a mechanism’s effect over a candidate purview is found at the minimum information partition (*MIP*) where the intrinsic difference *d*(*p*,*p_cut_*) is least. This yields the *integrated information* $\varphi_{MIP}$:

(2) $$\varphi_{MIP}\;=\;\underset{cut}{\min}d\left(p,\;p_{cut}\right)$$

In the case of the effect of mechanism CD over purview C^out^, the cut of D to C $(\frac{C^{c}}{C^{out}}\times \frac{D^{c}}{\left[\right]})$ is the MIP, yielding $\varphi_{MIP}\;=\;0.041$. 

Finally, the effect of a mechanism is the one that is *maximally irreducible*. Therefore, $\varphi_{MIP}$ is evaluated for all candidate purviews:

(3) $$\varphi_{effect}\;=\;\underset{purview}{\max}\varphi_{MIP}\left(purview\right)$$

In this case, CD^out^ = 00 is the maximally irreducible *effect*, with $\varphi_{MIP}$ = 0.081. 

The same procedure is followed on the cause side of the mechanism to reveal its maximally irreducible *cause—*in the case of mechanism CD, it is CD^in^ = 00, with $\varphi_{cause}\;$= 0.052 (^in^ for input state). Since mechanism CD has both a maximally irreducible cause and a maximally irreducible effect, it is said to specify a *cause-effect distinction*. By the same principle underlying the MIP, a mechanism cannot exist more than its weakest link, so the irreducibility of the distinction $CD^{c}/\langle CD^{in},CD^{out}\rangle$ is φ = min{0.052, 0.081} = 0.052. 

**Figure 4:**
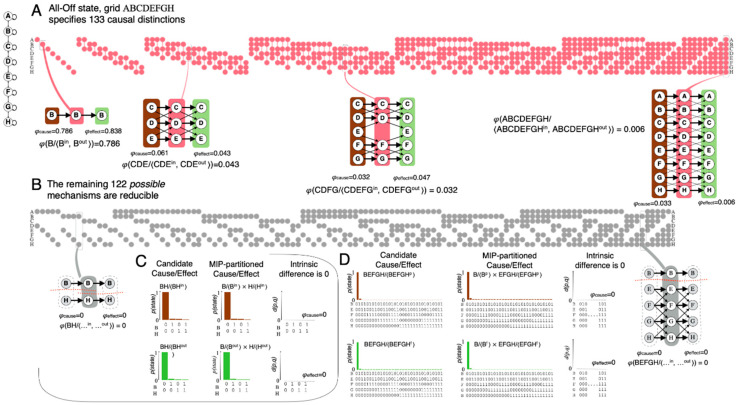
Causal distinctions specified by the 1D grid in the all-Off state. (**A**) The grid specifies 133 maximally irreducible distinctions. The pink array represents the mechanisms for each maximally irreducible distinction. Four examples are shown in DAG form. For each example, the mechanism, cause, and effect states are indicated by the colors of the units (in white for state 0). (**B**) The remaining 122 candidate mechanisms are reducible. (**C**,**D**) Two examples of reducible mechanisms are shown in DAG form.

The evaluation just described is repeated for the power set of all candidate mechanisms (here 255), yielding all the distinctions that compose the cause-effect structure specified by the system in its current state. In the all-Off state, the 8-unit grid specifies 133 irreducible distinctions (Figure 4A), while the other 122 candidate mechanisms are reducible (Figure 4B). A reducible mechanism is one whose cause and/or effect over any possible purview can be partitioned with no loss of intrinsic information (Figure 4C,D).

### 2.3. Relations: Maximally Irreducible Overlaps Among Cause-Effects

A distinction *links* a maximally irreducible cause with a maximally irreducible effect—it captures the difference made to a system by a mechanism in a state. While distinctions are the building blocks of a cause-effect structure, the way in which their causes or effects *overlap* within the system also makes a difference to a system. Therefore, to fully unfold the cause-effect structure of a substrate in a state, one must identify not only its specific cause-effects (distinctions), but also the specific way they overlap (relations). 

**Figure 5:**
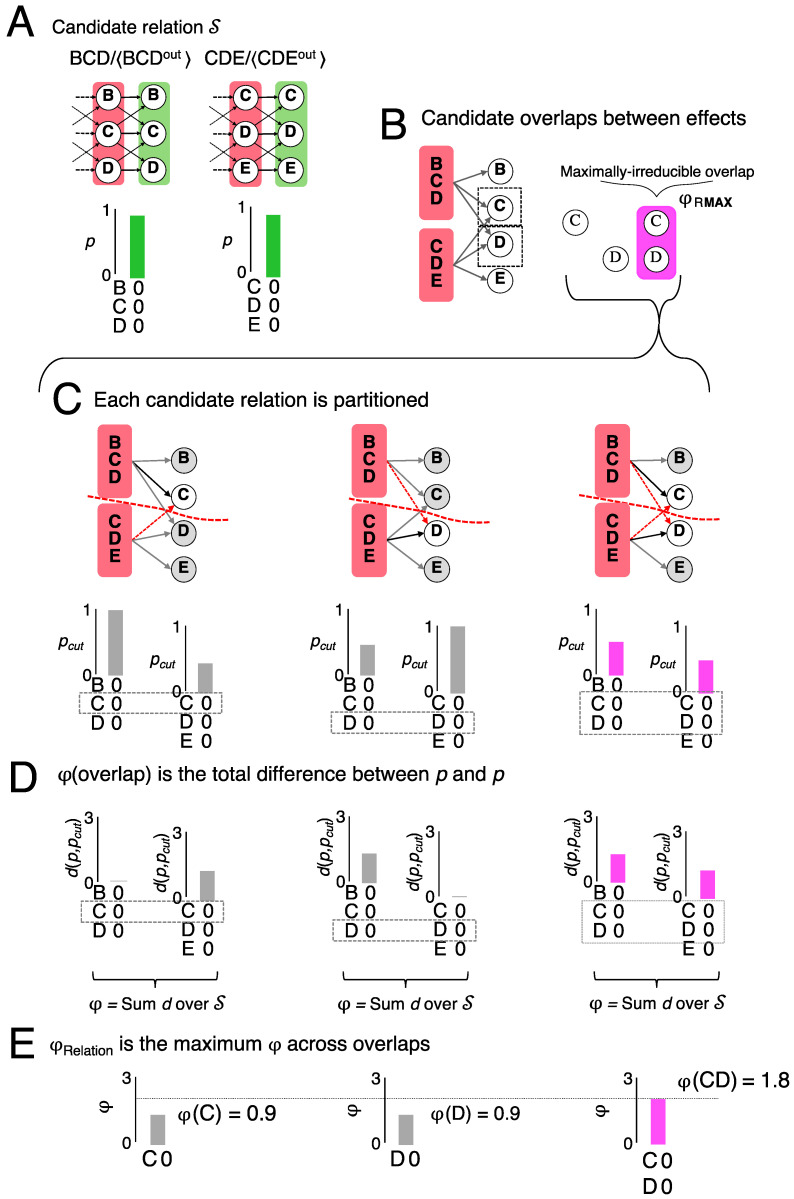
Characterization of a causal relation. (**A**) Two effects BCD/<BCD^out^> and CDE/<CDE^out^>. (**B**) These two effects are congruent over the units CD = [0, 0]. This allows for three candidate intrinsic overlaps, C = [0], D = [0], and CD = [0, 0]. (**C**) The candidate relation is partitioned so as to minimize the difference between the unpartitioned and partitioned causes and effects. (**D**) For each candidate overlap, the intrinsic difference is assessed on each effect involved, and these are summed over causes and effects. (**E**) The overlap CD = [0, 0] is maximally irreducible (in purple), so this is the overlap that exists intrinsically for these two effects, binding them together.

*Relations* are defined as maximally irreducible joint cause-effects that *bind* cause-effects within a cause-effect structure [12]. In what follows, we outline a procedure to characterize the relations specified by a substrate in a state. To exemplify, consider a candidate relation CD = 00 between the effect purviews specified by the distinctions $BCD^{c}/\langle BCD^{out}\;=\;000\rangle$ and $CDE^{c}/\langle CDE^{out}\;=\;000\rangle$ (Figure 5). The two effects are said to be *congruent* because their purviews overlap over the same units (CD) and they specify the same state (00). Congruence of the state of overlapping units is a necessary condition for a relation to exist. Two other candidate relations that are congruent between the two effects are over C = 0 or D = 0. By exclusion, the relation that actually exists between the two effects is the one whose congruence is maximally irreducible.

In general, we consider as *candidate overlaps* all possible subsets of units from the congruent intersection of the purviews of S. The irreducibility of every candidate overlap (*O_MAX_*) is then evaluated to identify the overlap that is maximally irreducible, which corresponds to the intrinsic relation between the causes and effects of S. As with causes and effects, a relation cannot exist more than its ‘weakest link’, so the irreducibility of a relation over a candidate overlap is found at the minimum information partition. Relation partitions are directly analogous to cause-effect partitions, but with the candidate set composed of a group of distinctions and their candidate overlap (rather than of a group of units-in-a-state and their candidate purview). In the example of Figure 5C, the MIP for the relation of effects $BCD^{c}/\langle BCD^{out}\;=\;000\rangle$ and $CDE^{c}/\langle CDE^{out}\;=\;000\rangle$ over the overlap *CD* = 00 is expressed as:

$$MIP\;=\;(\frac{BCD^{c}}{C^{out}}\times \frac{CDE^{c}}{D^{out}})$$

A relation partition resolves to partitions of each effect:

$$MIP\;=\;(\frac{BCD^{c}}{BC^{out}}\times \frac{\left[\right]}{D^{out}}),\;(\frac{\left[\right]}{C^{out}}\times \frac{CDE^{c}}{DE^{out}})$$

For each candidate overlap, the intrinsic difference is assessed at the MIP for each member of S:

(4) $$d_{O}\left(S_{i}\right)\;=\;\left|p\left(S_{i}\right)\log\frac{p\left(S_{i}\right)}{p{\left(S_{i}\right)}_{MIP}}\right|$$

The irreducibility of the candidate overlap *O* is the summed differences over S:

(5) $$\varphi \left(S/O\right)\;=\;\sum_{i}d_{O}\left(S_{i}\right)$$

Finally, the maximally irreducible overlap is the *O* that maximizes *φ*:

(6) $$\varphi_{Relation}\;,O_{MAX}\;=\;\underset{\left\{O\right\}}{\max,\;\mathrm{argmax}}\varphi \left(S/O\right)$$

For the relation of $BCD^{c}/\langle BCD^{out}\;=\;000\rangle$ and $CDE^{c}/\langle CDE^{out}\;=\;000\rangle$, the maximally-irreducible overlap happens to be at *CD* = 00.

The relations specified by a substrate can be characterized in various ways. The *order* of a relation is given by the number of distinctions involved. Thus, a relation between the cause and the effect of the same distinction is a 1st-order relation, between the effect of one distinction and the cause of another a 2nd-order relation, and so on. One can also characterize them by *type*, depending on how they bind the purviews of various distinctions. For example, a 1st-order relation within a single distinction is necessarily of type cause-effect, whereas 2nd-order relations between two distinctions can be of type cause-cause, cause-effect, effect-cause, and effect-effect. Depending on the number of purviews involved (causes or effects), relations can be characterized by their *degree* (as *k*-relations): relations between two purviews are 2-relations, among three purviews 3-relations, and so on. A cause-effect structure can be described as an *abstract simplicial complex* [13], where causes and effects are the vertices and relations are simplices: relations between pairs of causes and effects are 2-simplices (edges); relations between trios are 3-simplices (faces); and so-on. Relations are *complete* if they comprise all the causes and effects of the involved distinctions. For example, a 1st-order relation is necessarily complete (the 2-relation between cause and effect of the same distinction); a 2nd-order relation can be complete (if it is a 4-relation between causes and effects of two distinctions), and so on. Finally, relations can be *full* or *partial* depending on whether the purviews overlap fully or not.

### 2.4. Maximally Irreducible Cause-Effect Structure 

Once the distinctions and relations specified by a substrate in a state have been unfolded, integrated information theory requires that one evaluates whether the cause-effect structure they compose is itself maximally irreducible, compared to any other candidate substrate involving some or all of the same units. The irreducibility of a cause-effect structure is the overall integrated information across the substrate’s minimum cut (Φ or ‘big-Φ’, [6]). Here the calculation of Φ is omitted because this work focuses on assessing the quality rather than the quantity of experience.

## 3. Results

**Figure 6:**
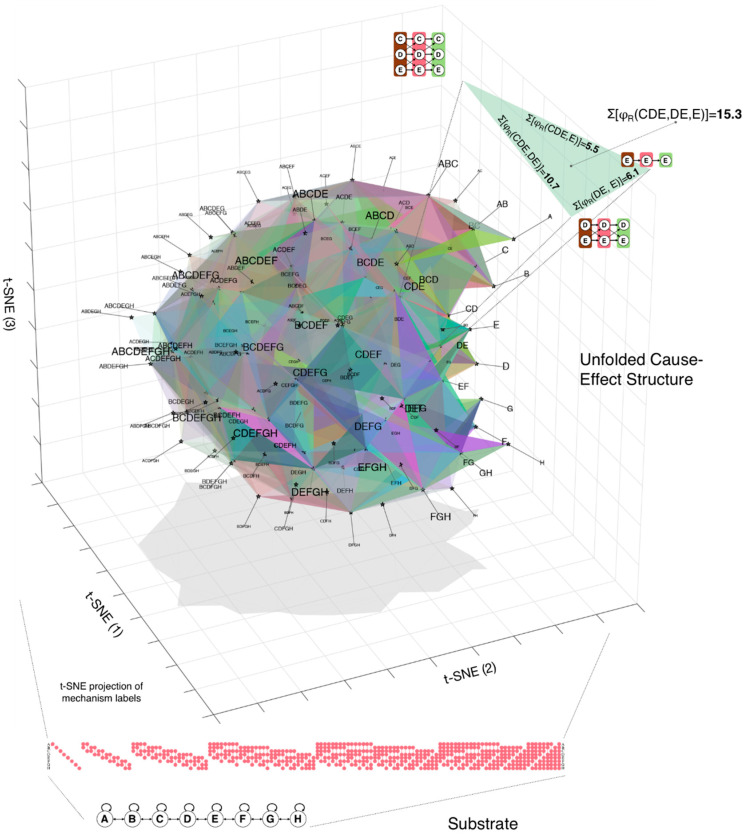
The cause-effect structure specified by the 1D grid in the all-Off state (only 2nd and 3rd order relations are displayed). All 133 distinctions specified by the grid have been projected to unique 3D coordinates using t-SNE over the mechanism labels. The labels at each vertex stand for the union of the cause- and effect- purviews for that distinction. Each edge between 2 vertices represents a complete 2nd-order relation. Each face among 3 vertices represents a complete 3rd-order relation. Labels font size is proportional to the sum of $\varphi_{Relation}$ for all the individual between-distinction relations. Face colors are assigned randomly. The inset at the upper right illustrates in more detail the relations among distinctions E, DE, and CDE.

The cause-effect structure unfolded from the 1D grid substrate in state all-Off described above is partly shown in Figure 6. Here the structure is illustrated as a simplicial complex. Each distinction has a 1st order relation (its cause relates to its effect), so it is represented by a single vertex; each edge represents a complete 2nd-order relation (a 4-relation between the causes and effects of both distinctions as well as the underlying 2- and 3-relations); each face represents a complete 3rd-order relation; higher-order relations are not shown. The coordinates of the vertices are a t-SNE [14] projection of the units that constitute the mechanisms and purviews of the distinctions. The t-SNE projection is a useful visualization tool since it happens to pick out two important symmetries of the grid’s intrinsic cause-effect structure: it orders distinctions by their cardinality (how many units they involve) and by the proximity of the grid units (A, B, C, …). Note that, in the case of the 1D grid, the same units tend to constitute the mechanism, cause, and effect of a distinction (Figure 4), simplifying the representation. Nevertheless, Figure 6 indicates that the cause-effect structure specified by a simple line-grid substrate in state All-Off is remarkably rich: consider that a 8-unit grid specifies 133 causal distinctions; between these there are 7976 2nd-order relations (‘edges’ in Figure 6) and more than 870,000 3rd-order relations (‘faces’ in Figure 6). 

In what follows, we assess whether this cause-effect structure can account for the fundamental spatial relation of extendedness, which encompasses connection, fusion, and inclusion of spots. We then examine spatial properties such as regions, locations, sizes, distances, and boundaries that derive from extendedness. Finally, we consider the effects of changing the state of some grid units, corresponding to spatial inhomogeneities, and of increasing their gain, corresponding to highlighting through spatial attention. We also consider whether these spatial properties can be accounted for not just at the level of individual distinctions and relations, but also at the level of compound distinctions and relations, which corresponds more directly to the coarse grain of introspective phenomenology.

A note on terminology and labels in this section: Unless otherwise specified, from here on ‘purview’ should be understood to refer to the combination of a distinction’s cause and effect purviews; ‘relations’ to bundles of relations (cause-cause, cause-effect, effect-cause, and effect-effect); ‘overlap’ to the combination of between-distinction relations (cause-cause overlap, cause-effect overlap and so-on); ‘*φ*_R_’ to the sum of $\varphi_{Relation}$ of the individual between-distinction relations (the cause-cause *φ* plus the cause-effect *φ* and so-on). Doing so simplifies the description of the cause-effect structure specified by a grid, taking advantage of its symmetries, primarily the fact that the cause and effect of most distinctions tend to be similar or the same. 

### 3.1. Spots

To bootstrap the process of accounting for phenomenal properties through physical properties, one must start somewhere. In the context of spatial experience, the place to start phenomenally is a *spot* picked out by introspection. One can easily distinguish any particular spot—*that* spot (Figure 1). As already pointed out, a spot can be anywhere within the canvas of space, ranging from the totality of space down to any of its points. A spot always overlaps itself, a property we call *reflexivity*. In terms of the cause-effect structure specified by a grid, the natural correspondent of a phenomenal distinction is a causal distinction: the mechanism specifying the distinction can be thought of as a ‘handle’ on the cause-effect structure, which ‘pulls’ with it other distinctions that have relations to its cause-effect. As shown in Figure 4 and Figure 6, the 1D grid specifies 133 distinctions whose cause-effects can pick out many subsets of units along the grid, ranging from the full set of units down to individual units. An important property of these distinctions is that each one has a 1st-order relation between its cause and its effect. This self-relation corresponds to the phenomenal property that a spot overlaps with itself (reflexivity). One can thus proceed to evaluate whether the relations among causal distinctions in the structure can account for the fundamental phenomenal property of *extendedness* which arguably characterizes spatial experiences. We define extendedness in terms of three relational structures—connection, fusion, and inclusion. 

#### 3.1.1. Connection

Phenomenally, one can check through introspection that, for any spot, one can always find spots with which it composes a *connection*: for any spot, one can find another spot that overlaps it partially and symmetrically, such that their intersection is also a spot; in turn, any spot is the intersection of two partially overlapping spots. Because two spots overlap over a spot, they overlap each other in the same way as a spot overlaps itself.

**Figure 7:**
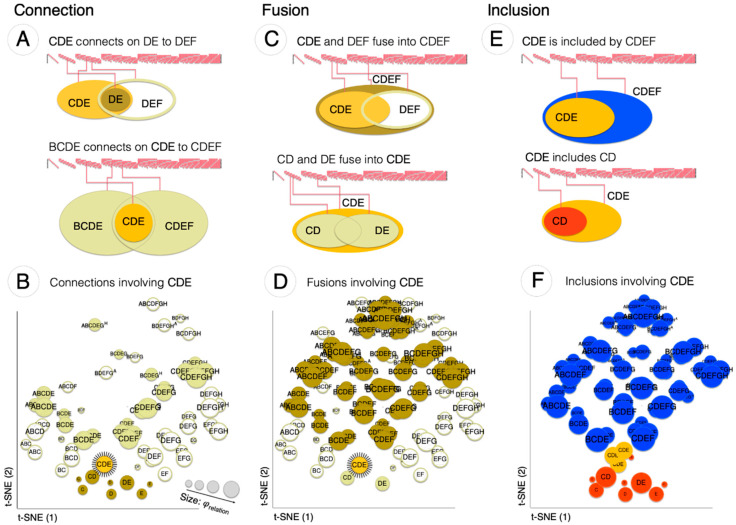
Extendedness of distinctions. (**A**) Grid distinctions are related by *connection*: for example, the distinction specified by mechanism CDE is connected to distinction DEF via distinction DE. CDE also connects the distinctions BCDE and CDEF. (**B**) A scatterplot of distinctions from the grid’s cause-effect structure. The axes are a 2D t-SNE projection of the distinction units. Each marker is labeled with the purview over which the distinction relates to itself. A grid distinction (gold) is connected with many other distinctions (open circles) through many other distinctions (olive), and it connects many other distinctions (pale brown). (**C**) Grid distinctions are related by *fusion*: for example, CDE is fused with DEF by CDEF. CDE also fuses CD and DE. (**D**) A grid distinction is fused with many other distinctions (open circles) into many other distinctions (olive), and it fuses many more (pale brown). (**E**) Grid distinctions are related by *inclusion*: for example, CDE is included by CDEF (in blue) and includes DE (in red). Some distinctions are mutually included (in gold). (**F**) Grid distinctions include (red) and are included by (blue) many other distinctions in the cause-effect structure. Note that CDE also mutually includes several other distinctions (BDE and CDF) that have the same cause-effect purviews, as represented in gold.

To account for phenomenal connection in terms of the cause-effect structure specified by a grid-like substrate, one needs to show that, for any causal distinction specified by the grid, one can find another distinction that is causally related to it in a way that satisfies connection, both ‘upward’ and ‘downward’. For example, consider the distinction CDE in Figure 7A. One can easily see that CDE is related to another distinction, in this case DEF, by 2nd-order relations among their causes and effects. These relations are complete because they involve all the causes and effects of the distinctions they relate. The completeness of the relation corresponds to reflexivity. Moreover, all these relations are partial (none involves all the units of either’s purview) and symmetric (cause-cause, effect-effect, cause-effect, and effect-cause, and so on). We also see that the intersection between CDE and DEF corresponds to a distinction (DE). Both CDE and DEF fully overlap DE, and any other distinction to which they both relate is also related to DE. DE is thus the *connection* of CDE and DEF. In turn, CDE is the connection between the partially overlapping distinctions BCDE and CDEF. Finally, Figure 7B shows that CDE is connected to many other distinctions besides DEF and is also the connection among many other distinctions within the system (Note: unless otherwise stated, when a set of units is referred to by a label such as CDE with no superscript, the label refers to the units that constitute the mechanism of the distinction).

#### 3.1.2. Fusion

One can also introspectively check that, for any spot, one can always find spots with which it composes a *fusion*: for any spot, one can find another spot that overlaps it partially and symmetrically, such that their union is also a spot; in turn, any spot is the union of two partially overlapping spots.

To account for phenomenal fusion in terms of the cause-effect structure specified by a grid-like substrate, one needs to show that, for any causal distinction specified by the grid, one can find other distinctions that are causally related to it in a way that satisfies fusion, both ‘upward’ and ‘downward’. Consider again the distinction CDE (Figure 7E). One can easily see that CDE is related, as before, to DEF, by 2nd-order relations among their causes and effects that are partial and symmetric. We also see that the union of CDE and DEF corresponds to a distinction (CDEF). CDEF fully overlaps both CDE and DEF, and any other distinction that overlaps CDEF also overlaps either CDE, DEF, or both. CDEF is thus the *fusion* of CDE and DEF. In turn, CDE is the fusion between partially overlapping distinctions CD and DE. Finally, Figure 7F shows that CDE fuses with many other distinctions besides DEF. 

#### 3.1.3. Inclusion

Finally, one can check through introspection that, for any spot, one can always find a spot that includes it (*inclusion up*) or is included by it (*inclusion down*). The phenomenal inclusion relation is reflexive, antisymmetric, and transitive, meaning that a spot includes itself, if it includes a second spot it cannot be included by it, and if the second spot includes a third spot, then the first spot includes the third.

To account for inclusion in terms of the cause-effect structure specified by a grid-like substrate, one needs to show that a causal relation that is reflexive, antisymmetric, and transitive is satisfied by most distinctions specified by the grid. Consider again distinction CDE and some of its causal relations (Figure 7C). Like all grid distinctions, CDE is related to itself through a 1st–order relation between its cause and effect (reflexivity). It also fully overlaps (down) the distinction DE, corresponding to a complete 2nd-order relations. These relations are antisymmetric, because CDE fully overlaps DE, but not the other way around. They are also transitive because, if there is a causal relation of full overlap (down) between DE and another distinction, such as D, then there is also a relation of full overlap (down) between CDE and D. The figure also shows that CDE includes (down) other distinctions besides DE and C, and is included (up) by many other distinctions within the structure (Figure 7D). The inclusion relation is denoted by the ≤ ≥ symbols. Specific instances of inclusion can be denoted by the labels inclusion-*down* (CDE ≥ DE), inclusion-*up* (CDE  ≤  CDEF) and *mutual*-inclusion (CDE = CDE).

#### 3.1.4. Extendedness: Grid-Like and Random-Like Graphs

**Figure 8:**
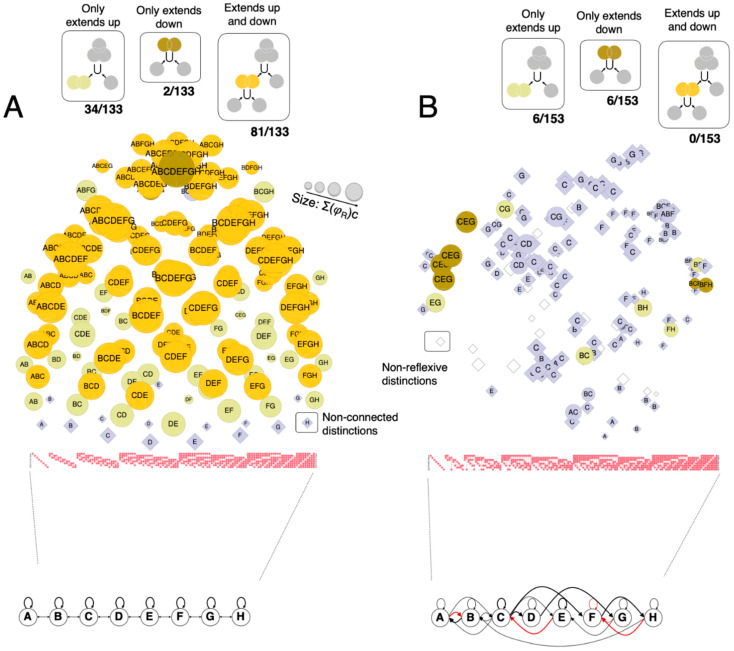
Extendedness of distinctions specified by the grid network and by a random network having the same number of units. A distinction is extended if its causal relations to other distinctions satisfy the properties of connection, fusion, and inclusion. (**A**) The grid and its cause-effect structure. Each marker in the t-SNE plot represents a single distinction, with a label identifying units in the cause-effect purviews. If units only appear in a cause purview they are shown in superscript, if only in an effect purview in subscript (this convention is clearer in panel (**B**)). Circle markers are distinctions that *connect* to other distinctions, while diamond markers are distinctions that do not. Of the 133 distinctions specified by the 1D grid, 81/133 extend both up and down to other distinctions (gold color); 36/133 extend only down (olive) or only up (pale brown); 16/133 are not connected (gray diamonds). Marker size is scaled to the total *φ*_R_ value of each distinction with the rest of the structure. (**B**) A random network with the same number of units as the grid network but with connections reassigned randomly specifies a cause-effect structure with 153 distinctions. 18 distinctions are not reflexive (white diamonds). None of the distinctions are fully extended.

Figure 8 summarizes which distinctions specified by the line-grid satisfy extendedness, characterized in terms of connection, fusion, and inclusion. In general, because of the highly symmetric connectivity among grid units, most distinctions satisfy connection (118/133). Furthermore, all distinctions are included (up and/or down) by a large number of other distinctions within the structure in a way that accords with reflexivity, antisymmetry, and transitivity. Reflexivity holds across the structure because the cause and effect of grid mechanisms are most often identical (115 out of 133) and otherwise very similar (16 with cause and effect differing by a single unit, 2 distinctions by 2 units). Most distinctions are extended (117/13): they satisfy fusion in addition to connection and inclusion. One distinction, the *total* distinction (ABCDEFGH), is purely extended down, because it includes the entire grid and is only a fusion of other distinctions; the remaining 16 distinctions are reflexive and yet neither extended up or down, because they have 1-unit purviews that neither connect to nor include down any other distinction. Given that most distinctions and relations specified by a grid-like substrate, except for total and point distinctions, satisfy extendedness, we call the cause-effect structure specified by a grid-like substrate an *extension*.

By way of comparison, Figure 8B summarizes which distinctions satisfy extendedness—connection, fusion, and inclusion—for a ‘random’ network (Figure 8B), constituted of the same number of units and connections as the line-grid, but with connections scrambled quasi-randomly (allowing some to become negative, indicated in red). The cause-effect structure specified by the random network has 153 distinctions, 20 more than the grid, but of these only one is connected both up and down, none are fused up and down, and therefore none are extended. Self-relations in the random network are mostly over single units, as shown in Figure 8B, and 18 distinctions have no self-relation at all. Similar results were obtained for other “random” networks (not shown). 

#### 3.1.5. Regions

**Figure 9:**
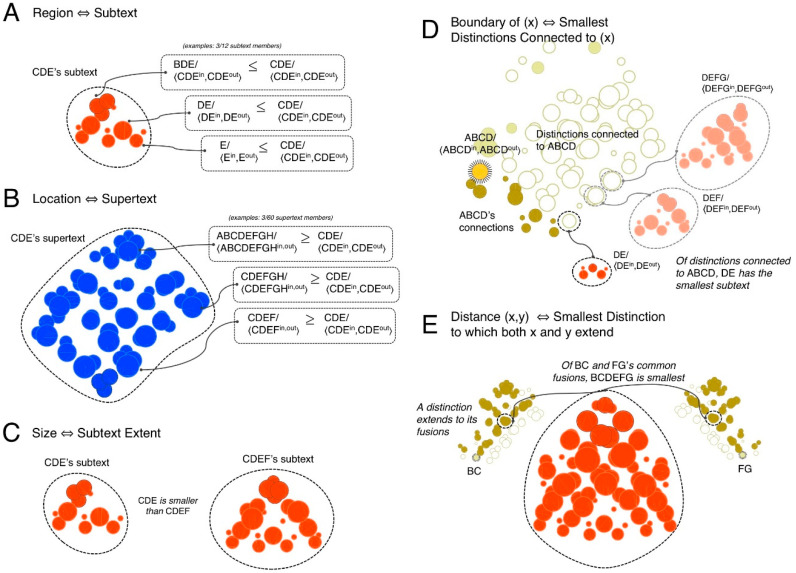
Properties of the distinctions specified by the 1D grid. (**A**) The *subtext* of a distinction is the set of other distinctions it down-includes (in orange). Three examples of down-inclusion by CDE are shown. (**B**) The *supertext* of a distinction is the set of other distinctions it up-includes (in blue). Three examples of up-inclusion by CDE are identified. (**C**) The size of a distinction is given by the number of distinctions in its subtext. (**D**) The boundary of a distinction is the set of the smallest distinctions that are connected to it (open circles). Here, the boundary of ABCD (region shown in orange) is just DE. Two larger distinctions connected to ABCD are shown for comparison (DEF and DEFG). (**E**) The distance between two distinctions is the smallest distinction to which they both extend up (their least fusion; fusions shown in olive). Here, BC and FG extend up to BCDEFG.

Several properties of spatial experience can be understood as derivable from extendedness. Introspectively, the *region* covered by a spot can be thought of as the set of all the spots it fully overlaps. In the cause-effect structure specified by a grid, a region corresponds to all the distinctions that are included down by a particular distinction—its *subtext* (Figure 9A, in orange). More strictly, it corresponds to those down-included distinctions that decompose it recursively (CDE decomposes to CD and DE). This stricter criterion excludes the 1st-order distinctions ($A^{c}/\langle A^{in},A^{out}\rangle$,$\;B^{c}/\langle B^{in},B^{out}\rangle$, and so on).

#### 3.1.6. Locations

Introspectively, the *location* of a spot can be thought of as the set of all the spots that fully overlap that spot, constraining its place within the totality of the space. A helpful analogy is a geographical address: the location of a place is given by a set of larger places that include it. In the cause-effect structure specified by a grid, a location corresponds to all the distinctions that include up a particular distinction—its *supertext* (Figure 9B, in blue). More strictly, it corresponds to those up-included distinctions to which it extends recursively (as CDE extends to CDEFG, which extends to ABCDEFGH).

#### 3.1.7. Sizes, Boundaries, and Distances

Ideas about space are often built around notions like size and distance as defined operationally from an extrinsic, ‘objective’ (intersubjective) perspective. However, these notions are ultimately based on the intrinsic, subjective perspective of a conscious experiencer. For example, the phenomenal *size* of a spot can be characterized introspectively by the number of spots it includes down (the size of its region), or more precisely as the number of ways the spot can be decomposed down to points. In the cause-effect structure specified by a grid, the size of a distinction can be defined as the number of distinctions it fully includes (the size of its subtext), or more precisely as the number of ways the distinction can be decomposed down to distinctions that are purely extended up (Figure 9C). 

Another property of spatial experience is the *boundary* of a spot. For any spot, we can introspect that it has a boundary, which can be thought of as the set of the *smallest* spots that are connected to it. In the cause-effect structure, the boundary of a distinction corresponds to the set of the smallest distinctions that are connected to it (Figure 9D). Also, just as a space itself has no boundary, a distinction may have no connections if it is purely extended down (the ‘total distinction’, e.g., ABCDEFGH). The example of Figure 9D captures this aspect of boundaries in a space: the distinction ABCD has a region extending from A to D, but its only boundary spot is DE: ABCD has no boundary on its ‘A side’.

Finally, the phenomenal *distance* between *any* two spots can be thought of as the smallest spot they both extend (up) to, i.e., their *least fusion*. In terms of the cause-effect structure specified by a grid, the distance between two distinctions corresponds to the subtext of the smallest distinction to which they both extend (up) (Figure 9E). Importantly, a distance can be defined for *any* pair of distinctions within the structure, in accordance with intuitions about space.

Together, the set of relations that bind a given distinction to other distinctions within the cause-effect structure form its *context*. If a mechanism is a ‘handle’ on the cause-effect structure, the context is what one gets when the handle is ‘pulled’. The context of a distinction is analogous to the star of a vertex in a simplicial complex [13]. 

### 3.2. Inhomogeneities

**Figure 10:**
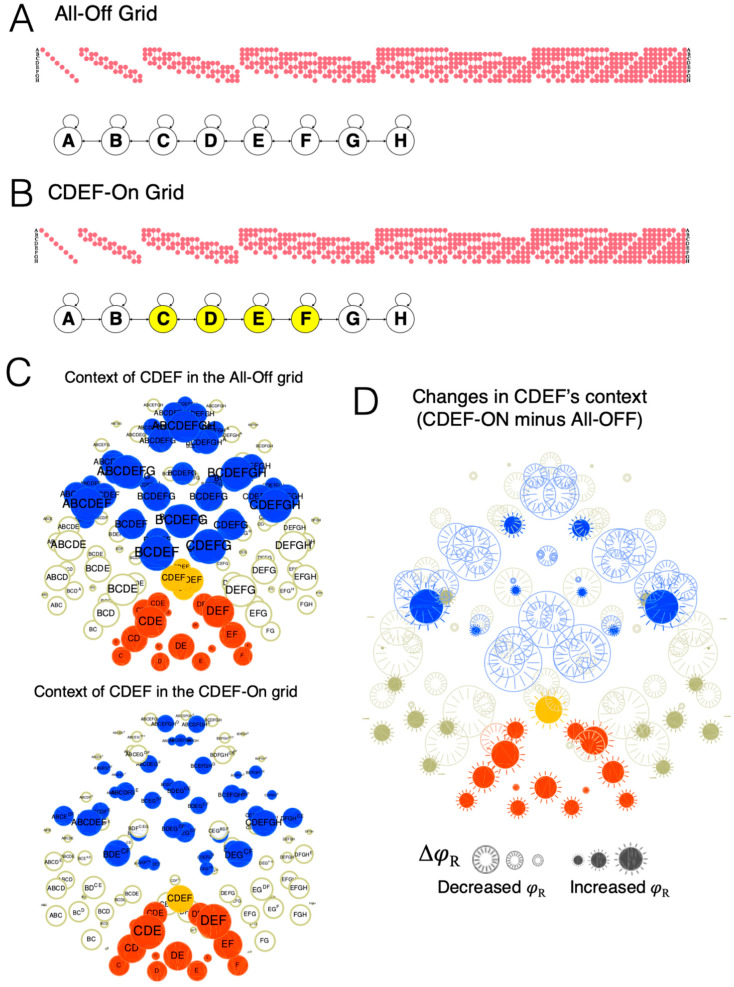
Inhomogeneities. (**A**) 1D grid in the all-Off state. (**B**) The same grid in an inhomogeneous state, with CDEF-On: The cause-effect structure remains similar to the All-Off state, with the same mechanisms existing in both states. (**C**) As shown by its context, the distinction CDEF exists in both the all-Off and CDEF-On states. The relations between CDEF and the rest of the structure are qualitatively similar for both states. Colors indicate connection (open pale-brown symbols), inclusion up (blue), inclusion down (orange), and self-inclusion. (**D**) The difference in the context of CDEF for the all-Off and CDEF-On states (*φ*_R_[all-Off] − *φ*_R_[CDEF-On]). Solid symbols represent increased *φ*_R_, hollow symbols represent decreased *φ*_R_. The subtext of CDEF is generally more irreducible (higher *φ*_R_) in the CDEF-On state, whereas its supertext and its connections are generally more reducible (lower *φ*_R_).

Introspectively, spatial *inhomogeneities* are local disruptions in extendedness. For example, one or more spots may ‘stand out’ from the rest of space, say bright stars in the otherwise dark sky, even though the overall, extended fabric of space is preserved. Typically, an inhomogeneity is associated with a difference in local qualities, such as colors. However, there is a purely spatial aspect to an inhomogeneity, since the same inhomogeneity can be associated, at the same location, with a boundary between a bright and a dark region, a red and a blue region, and so on. Figure 10 shows the consequences of introducing an inhomogeneity by turning On a few units (CDEF), as would be the case if they were activated by a stimulus. Compared to the cause-effect structure specified by a grid with all units Off, many relations are reduced in their *φ*_R_ value (indicated by the ‘hollow’ symbols), especially the supertext of CDEF. On the other hand, other components of the structure are enhanced (indicated by the ‘solid’ symbols), especially the subtext of CDEF and that of other distinctions included by it {CDE, DEF, CD, DE, EF}. Some distinctions to which CDEF is connected are enhanced, in particular its two boundary spots BC and FG. Importantly, the overall cause-effect structure is still generally extended (75/133 distinctions are extended up and/or down). Translating back to phenomenology, the overall effect of turning on a patch of units is that the region they specify is made more salient, its location within the rest of space less so, while the experience remains altogether spatial. 

It is important to note that the consequences of an inhomogeneity in the state of some units can depend strongly on their activation function. For example, if the strength of connections among active units were to increase above their baseline level, the *φ* value of most distinctions and relations they specify may increase as well.

### 3.3. Attentional Highlighting

Introspectively, the *spotlight* of attention can shift from one location to another as well as expand or contract [15,16]. Phenomenally, the attended region of space appears to be *highlighted*—as if more of the experience were concentrated there—at the expense of the rest of the canvas.

**Figure 11:**
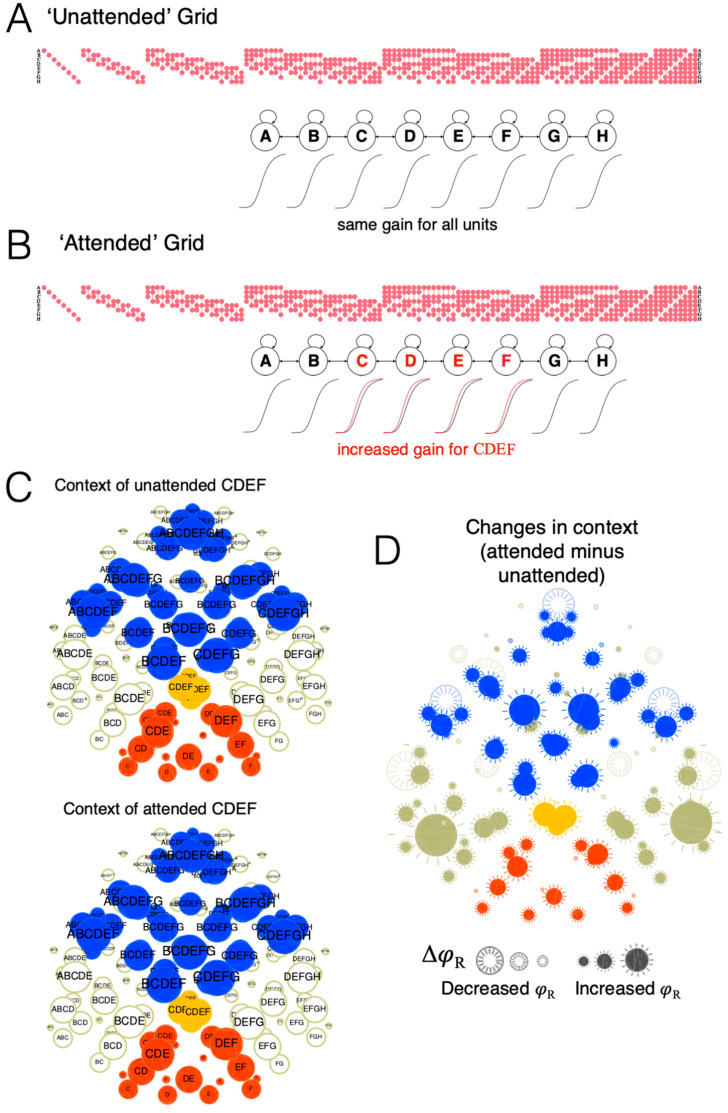
Attentional Highlighting. (**A**) 1D grid in the ‘unattended’ all-Off state. (**B**) The 1D grid in the ‘attended’ all-Off state: to simulate the ‘spotlight’ of attention, units CDEF have increased response gain (red lines). (**C**) The distinction CDEF exists for both the unattended and attended grids. The relations between CDEF and the rest of the structure are qualitatively similar for both the unattended and attended grids. Colors are as in Figure 10. (**D**) The difference in the context of CDEF for the attended and unattended grids (*φ*_R_[unattended] − *φ*_R_[attended]). Symbol scaling is greater by a factor of 1.5 relative to Figure 10D. The context of CDEF is generally more irreducible when the gain of CDEF is increased by the ‘spotlight’ of attention.

This local highlighting has a direct correlate in the cause-effect structure of an attended spot within a grid, assuming a simple model of spatial attention. Spatial attention involves an increase in the response gain of neurons in the selected region of space [17,18,19], which was simulated by increasing the response gain of spotlighted units in the grid. In Figure 11, the ‘unattended’ grid, with all units Off, is the same as in all previous examples (with threshold *z* = 0.25). To simulate and ‘attended’ spot, the gain of units CDEF was slightly increased (their *z* reduced by 10% to *z* = 0.225). The unfolded cause-effect structure reveals an enhancement of many relations, especially those belonging to the context of distinctions CDEF, CDE, and DEF. Again, the overall cause-effect structure is still extended (112/133 distinctions are extended up and/or down). Translating back to phenomenology, the overall effect of increasing the gain of a subset of units is that the spot they specify is highlighted.

### 3.4. Compound Distinctions and Relations

The previous sections provided evidence for a close correspondence between phenomenal properties of spatial experience and physical properties of a substrate at the level of the individual distinctions and relations that compose the cause-effect structure it specifies. However, the section on attentional highlighting also showed that, when we introspect and employ the spotlight of attention to increase the gain of a subset of units, we highlight not just individual distinctions and relations, but rather *groups* of distinctions and relations, composed of many distinctions and their contexts. For example, as illustrated in Figure 11, attending to a subset of units within the grid (CDEF) affects not only the distinction CDEF, but all the included distinctions CDE, DEF, CD, DE, EF, CE, DF, C, D, E, F, together with their contexts. Accordingly, the combination of all the distinctions picked out by CDEF is called a *compound distinction*, and the their combined context is called a *compound context* (within which one can distinguish, as usual, connections, fusions, and inclusions).

Strictly speaking, then, it is at the level of compound distinctions and contexts that we can handily employ introspection to establish a correspondence between phenomenal and physical properties, including spots and the fundamental property of spatial extendedness. Even so, it can be shown that, if a phenomenal spot is taken to correspond to a compound distinction, extendedness percolates from the level of individual distinctions and contexts to that of compound distinctions and contexts. Specifically, the properties of connection, fusion, and inclusion among spots hold for contexts of distinctions, which in turn implies that they must also hold for compound distinctions and compound contexts. Thus, when a distinction is connected to another, their compound contexts must also be connected—in particular their compound subtexts. When two distinctions are fused, their compound contexts must overlap in a fusion. Finally, when a distinction is included by another, the former’s compound context must be fully overlapped by the latter’s. Similar considerations apply to regions, locations, sizes, distances, and boundaries.

### 3.5. A 2D Grid

**Figure 12:**
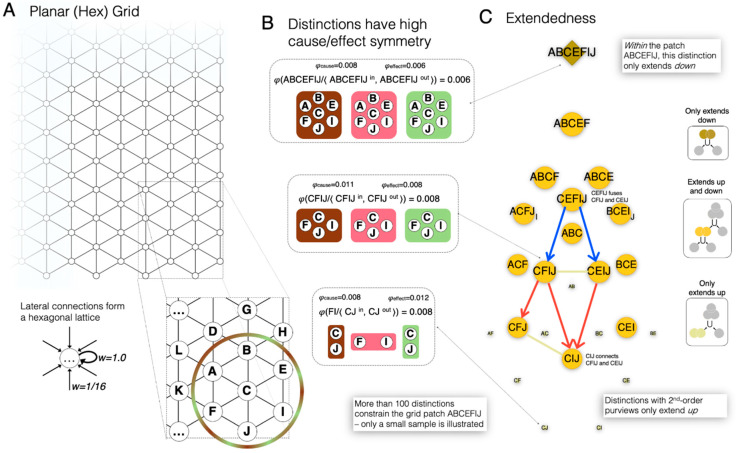
Extendedness in a 2D grid. (**A**) A hexagonal 2D, planar grid that works in a similar way to the 1D line-grid of Figure 2. Each unit pools six lateral inputs and a self-input, determining the probability of its turning On in the next time step. Here all units are Off. A small corner of the grid is selected for analysis (brown/green circle). (**B**) A representative sample of distinctions that have cause-effect purviews within the selected corner of the grid. For all three distinctions, the mechanism specifies causes and effects over a similar subset of units and states, as is the case with the line-grid. (**C**) The symmetric arrangement of the causes and effects ensures that the causal relations among the distinctions satisfy extendedness (connection, fusion, inclusion). A sample of distinctions satisfying extendedness is shown with the same conventions as in Figure 7 and Figure 8. Axes in this plot are not derived from t-SNE but are set to reflect the geometry of the grid.

The examples in this section have focused on a small 1D line-grid network because it allowed us to unfold its cause-effect structure in its entirety. Even in this case, the cause-effect structure turns out to be composed of a remarkably large number of distinctions and relations. Of course, our experience of space, which motivated the characterization of extendedness, is (at least) 2D-extended, rather than 1D-extended. Furthermore, its neural substrate is likely constituted by 2D-grids, rather than 1D grids. Nevertheless, the organizational principles that make a cause-effect structure extended generalize from 1D to 2D substrates, because they are determined by the near-neighbor arrangement of the connectivity. This point is illustrated in Figure 12. Figure 12A shows a 2D, planar ‘hex’ grid, in which each unit is connected to itself and to six neighbors arranged according to the vertices of a hexagon. Weights and activation rules are similar to the line network of Figure 2, with parameters adjusted to account for the increase number of connections. Specifically, the lateral connections are weaker, with *w* = 0.0625; the Naka-Rushton exponent is shallower, with *n* = 3; and the threshold is higher, with *z* = 0.4125. As in the 1D case, every contiguous patch of the 2D grid specifies a causal distinction, three of which are illustrated in Figure 12B. As with the 1D grid, each distinction specified by the 2D grid specifies a cause and effect over a similar subset of units. Furthermore, the distinctions specified by the 2D grid are related in a way that satisfies extendedness (connection, fusion, inclusion) in a way similar to the 1D grid (Figure 12C). 

**Figure A1:**
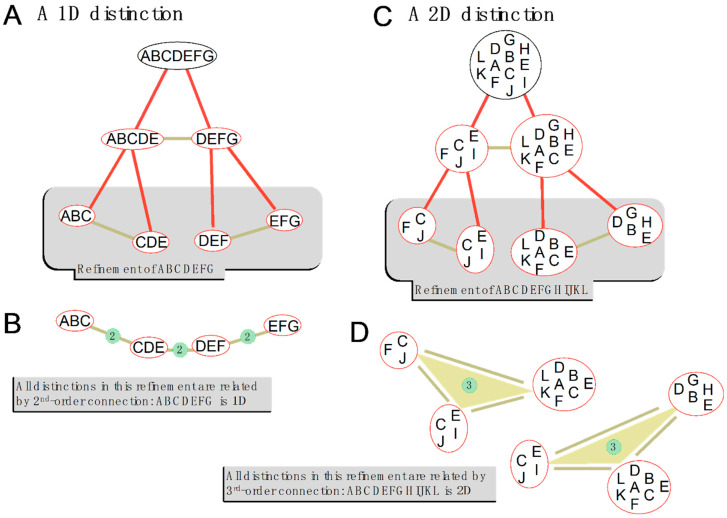
The causal properties underlying the dimensionality of spatial structures. (**A**) An idealized 1D distinction ABCDEFG is refined through recursive fusion-down to the distinctions {ABC,CDE,DEF,EFG}. (**B**) Note that, in this refinement of ABCDEFG, every distinction is related to some other distinction by a 2nd-order connection relation. For 1D distinctions in general, every refinement is such that a distinction is always related to some other distinction by a 2nd-order connection relation; furthermore, there is always at least one refinement with no connection relations of order > 2. These two constraints characterize distinctions belonging to a 1D space. (**C**) An idealized 2D distinction ABCDEFGHIJKL is refined to the distinctions {CFJ, CEIJ, ABCDEFLK, BDEGH}. (**D**) Note that, in this refinement of ABCDEFGHIJKL, every distinction is related to some other distinction by a 3rd-order connection relation. For 2D distinctions in general, every refinement is such that a distinction is always related to some other distinction by a 3rd-order connection relation; furthermore, there is always at least one refinement with no connection relations of order >3. These two constraints characterize distinctions belonging to a 2D space. The dimensionality of extendedness corresponds to the prescribed order of the connection relation minus 1 and is a close analogue of the Lebesgue covering dimension of classical topology, as described in Appendix C.

The difference between the cause-effect structures specified by 1D and 2D grids resides instead in the dimensionality of extendedness. Appendix A Figure A1 shows a *refinement* of a distinction as a recursive decomposition of a ‘top’ distinction, based on the three relations that characterize extendedness. A decomposition yielding a refinement proceeds through fusion-down relations (starting with a distinction, and then finding two distinctions that it fuses), with the requirement that the smallest distinctions included by the ‘top’ distinction are conserved throughout the decomposition (analogous to the notion of ‘open cover’ from topology; see Appendix C). In the 1D case, every refinement is such that any of its distinctions is always related to another by a 2nd-order connection relation; furthermore, there must be at least one refinement with no connection relations of order >2. In the 2D case, every refinement is such that any of its distinctions is always related to *two* others by a 3nd-order connection relation; furthermore, there must be at least one refinement with no connection relations of order > 3. The dimensionality of extendedness, corresponding to the prescribed order of the connection relation minus 1, is a close analogue of the Lebesgue covering dimension of classical topology, as described in Appendix C.

## 4. Discussion

We have employed the tools of integrated information theory (IIT) to show that basic phenomenal properties of spatial experiences—what makes space feel the way it does—can be accounted for, in a principled manner, by physical properties of a grid-like substrate, namely by the cause-effect structure it specifies. The one-to-one correspondence between phenomenal and physical properties outlined above represents a first attempt to deploy the theory to account for the quality of experience. Previous theoretical work focused primarily on the general requirements for a physical substrate of consciousness [6,7,20] and on the grain of its constituent units [20,21]. Complementary empirical work assessed the prediction that the breakdown of consciousness should be associated with a breakdown of the brain’s capacity for integrated information [22,23]. 

As a first test case, we chose the phenomenology of space because spatial experiences are pervasive and more penetrable by introspection than other aspects of consciousness. Moreover, the physical substrate of spatial experience may be comparatively easy to characterize in neural terms. Below we briefly consider some of the difficulties that stand in the way of a proper appreciation of a scientific account of spatial qualia; the proposed correspondence between the phenomenal properties of spatial experiences and physical properties of grid-like substrates, as given by the cause-effect structures they specify; the evidence that grids in the brain may constitute the substrate for spatial experience; some ensuing predictions; and the inadequacy of approaches that cannot account for the way spatial experience feels.

### 4.1. The Problem of Spatial Qualia

The account of spatial experience investigated in this paper must contend with deep-seated assumptions about the nature of both the phenomenal and the physical. A first problem has to do with spatial experience itself. Much work has been devoted to understanding how external physical space—the space ‘out there’—is represented in the brain in a way that accounts for spatial functions and behaviors (see Note 6 in Appendix B). The way space is experienced within consciousness has received much less thought (see Note 7 in Appendix B). However, it is essential to realize that the subjective feeling of extendedness—what it is like to experience the vast canvas of the sky—is just is as qualitative and in need of explanation as that of color or pain. But it is easier to tackle, because of our ability to penetrate introspectively some aspects of the structure of spatial experience, an ability we mostly lack for color or pain (see Note 8 in Appendix B).

Second, one must realize that there is much more than meets the eye to a spatial experience, even a seemingly ‘simple’ one. Consider the experience of a blank canvas. The two words ‘blank canvas’ seem sufficient to convey the content of the experience to other human beings. However, this brevity in communication is only possible because it presupposes that other humans already know what space feels like. Things change if one is to characterize what an experience of extendedness feels like without taking space as given. To do so, one needs to identify the phenomenal components that make a canvas feel extended. As suggested here, these components are a large number of distinctions, corresponding to experienced spots; and an immense number the relations that bind them into a single phenomenal structure according to connection, fusion, and inclusion, corresponding to the feeling of extendedness. Phenomenal regions, locations, sizes, boundaries, and distances can then be described as sub-structures within this overall organization of distinctions and relations. 

Third, one must realize that, to account in physical terms for *spatial experience as such*, it is not enough to account for how the brain represents external space and performs spatial functions. Instead, one should strive to provide a one-to-one correspondence between the phenomenal distinctions and relations that compose the experience and the causal distinctions and relations that compose the cause-effect structure specified by a physical substrate (see Note 9 in Appendix B). Otherwise, the structure of experience remains completely unexplained—associated to its physical substrate in the brain in a way that seems utterly mysterious as well as arbitrary. 

Fourth, one must realize that there is much more than meets the eye (and the hand) to a physical substrate. We typically think of a physical substrate, say a neural network, as consisting of nothing but neurons and their connections. By observing and manipulating the units of the network we can understand how it does what it does and ultimately predict its behavior. However, as illustrated here with a small grid of binary units, to properly characterize the cause-effect powers of a substrate one must unfold its cause-effect structure in full, making explicit all the causal distinctions and relations it specifies. Only then can one attempt to establish a correspondence between what exists in phenomenal and physical terms. 

### 4.2. The Causal Properties of Grid-Like Physical Substrates and Their Correspondence to the Phenomenal Properties of Spatial Experiences 

As stated in the Introduction, according to integrated information theory every experience has five essential properties: intrinsicality (existing for the subject of experience); composition (being structured by phenomenal distinctions and their relations); information (being the specific way it is); integration (being irreducible); and exclusion (being definite). Spatial experiences satisfy these properties and appear to add further properties that make them characteristically spatial (Figure 1A). To recapitulate, the canvas of space, even in the absence of any object, is composed of a vast number of distinctions (spots, Figure 1B) that are bound to each other by characteristic relations (extendedness, Figure 1C). Specifically, one can determine introspectively that every spot within the canvas overlaps with itself; it connects to some other spot and is the connection of two other spots; it fuses with and is the fusion of other spots; and it includes (down) or is included (up) by some other spot. The only exceptions are the total spot, which only extends down to other spots, and the points, which only extend up to other spots. Other phenomenal features of spatial experience can be thought of as deriving from the fundamental property of extendedness (Figure 1D): the region of space picked out by a spot, its location in space, its size, boundary, and distance from other spots. Finally, one can experience regions of space that stand out from the rest of space due to local inhomogeneities, or because they are highlighted by attention.

Integrated information theory then claims that every property of an experience must be accounted for by a property of the cause-effect structure specified by a physical substrate in a particular state. What kind of physical substrate could specify a cause-effect structure that can account not only for the properties that are true of every conceivable experience (intrinsicality, composition, information, integration, and exclusion), but also for the properties that make an experience characteristically spatial? After illustrating how the tools of the theory can be employed to fully unfold the cause-effect structures specified by a simple substrate, we analyzed the cause-effect structure specified by a grid-like substrate, primarily a small 1D grid. The Results section indicates that the cause-effect structure specified by the grid, but not by a random network, can in principle account for many properties of spatial experiences. 

As shown in Figure 6, the cause-effect structure specified by a 1D grid is composed of a large number of causal distinctions and a very large number of relations that bind them in a characteristic way. In the case of the grid, the existing distinctions (those that are maximally irreducible) specify causes and effects that overlap over individual units (1st-order distinctions); over pairs of adjacent or nearly adjacent units (2nd-order), over triplets of adjacent or nearly adjacent units (3rd-order); and so on, up to a highest-order distinction that specifies a symmetric cause and effect over all the units of the grid. As shown in Figure 12, the organization of distinctions according to adjacency also applies to a 2D grid. Note that, for each distinction specified by a grid, the cause overlaps with the effect, yielding a 1st-order complete relation - a self-relation characterized by reflexivity.

**Figure 13:**
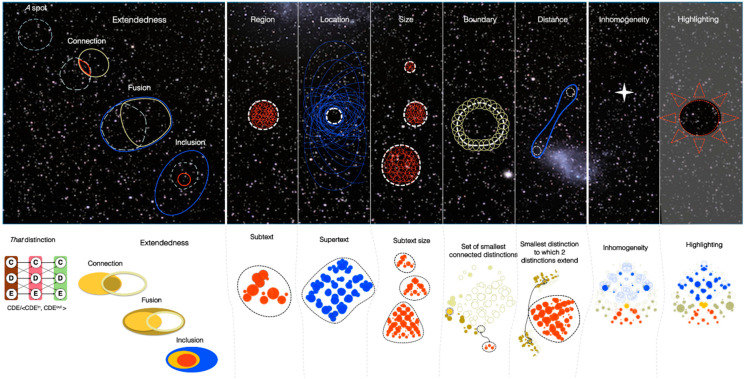
Correspondence between phenomenal properties of spatial experience and physical properties of a grid-like substrate, given by the cause-effect structure it specifies. From left to right, phenomenal properties are listed above their proposed physical correspondent. *That* particular spot, which overlaps itself, is picked out by a particular causal distinction, whose cause overlaps its effect (reflexivity). A spot feels *extended* in that connects, fuses, and includes other spots; correspondingly, distinctions specified by a grid satisfy extendedness through relations of connection, fusion, and inclusion with other distinctions. Other properties of spatial experience, such as the region and location of a spot correspond respectively to the subtext and supertext of the corresponding distinction; the size of a spot to the number of distinctions in its subtext; the boundary of a spot to the set of the smallest distinctions connected to its corresponding distinction; its distance from other spots to the size of the smallest distinction to which their corresponding distinction extend up. A spatial inhomogeneity—here a bright star on a dark background—corresponds to a local warping in the cause-effect structure; an attended spot corresponds to a local highlighting.

The adjacency among units is apparent to an external observer who is already endowed with the ability to recognize this kind of ordering. However, from the intrinsic perspective of the system itself, the ordering must be established by the intrinsic organization of the causal relations among the distinctions specified by its mechanisms. As shown here, most distinctions specified by the grid are bound to other distinctions by causal relations that conform to extendedness (Figure 13): each distinction connects to some other distinction that overlaps it partially and symmetrically; it likewise fuses with or is the fusion of other overlapping distinctions; and it includes or is included by other distinctions. The only exceptions are the total distinction that only extends down to other distinctions, and the point distinctions that only extends up to other distinctions. Altogether, the extendedness relations yield a partial ordering of distinctions that corresponds precisely to the partial ordering of spots in spatial experience. Again, the relations that characterize extendedness also apply to the cause-effect structure specified by a 2D grid, with the difference that distinctions extend in 2 dimensions, rather than 1 dimension, corresponding to a Lebesgue dimension of 3 and 2, respectively (Appendix C). 

Other phenomenal properties of spatial experience that are derivable from extendedness find a correspondent in the cause-effect structure specified by the grid: the region of space picked out by a spot corresponds to the set of all distinctions fully included by a given distinction (its subtext); the location of a spot to the set of all distinctions that fully include a given distinction (its supertext); the size of a spot to the number of distinctions fully included by a given distinction; the boundary of a spot to the set of smallest distinctions that are connected to a given distinction; the distance between any two spots as the smallest distinction to which two distinctions extend (up) (see Note 10 in Appendix B). 

Finally, local inhomogeneities that emboss the canvas of phenomenal space, such as stars in the night sky, can be accounted for by local inhomogeneities in the cause-effect structure. Attentional highlighting, which similarly makes some spots stand out from the rest of space, can be accounted for by the selective enhancement of some distinctions and relations.

**Figure 14:**
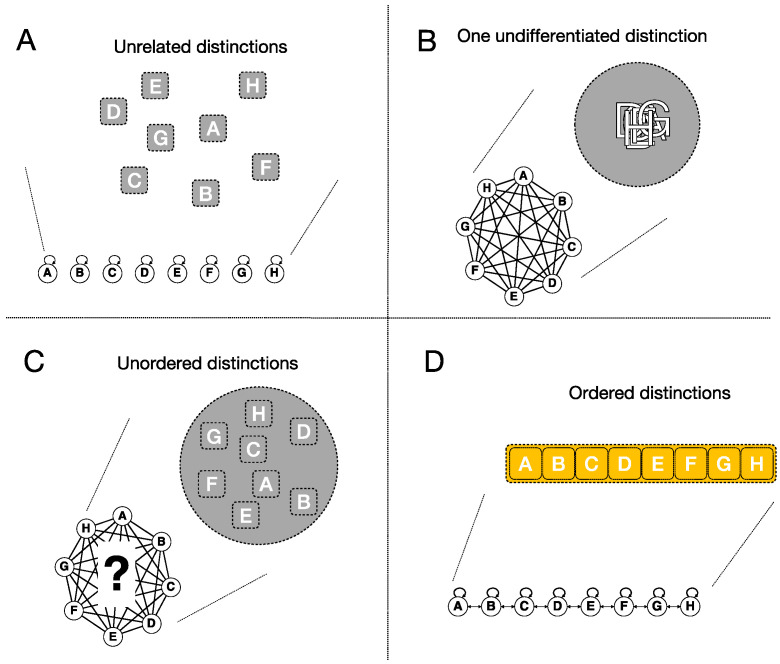
Space in a nutshell (see text for description).

We end this section with an example that highlights, in a nutshell, some minimal requirements that a cause-effect structure must satisfy to account for experiencing space, again in a simple 1D case (Figure 14). Figure 14A–C illustrates reduced substrates whose cause-effect structures lack critical ingredients. For instance, if only the bottom-order cause-effects were specified (Figure 14A), it would not be possible to experience them as composing a larger distinction, hence none of them could feel extended (in fact, in this case they would not even be integrated). If only the top-order cause-effect were specified (Figure 14B), it would not be possible to experience that phenomenal distinction as composed of finer distinctions, hence it could not feel extended but at most as a single undifferentiated ‘lump’. If both the top- and bottom-order cause-effects were specified, but no purviews and relations in between, the bottom-order purviews would be related to the top one, but they would not be ordered with respect to each other, hence the structure could not feel spatial either (Figure 14C) (see Note 11 in Appendix B). In short, experiencing the extendedness that characterizes space requires a substrate that specifies a large number of causal distinctions and relations that bind them in an appropriate order, which corresponds to extendedness (Figure 14D). A grid offers exactly this kind of substrate. Accordingly, we called the cause-effect structure specified by a grid-like substrate an *extension*.

### 4.3. Some Outstanding Issues 

At this stage, we have not explicitly addressed several additional properties associated with phenomenal space, beginning with the phenomenal experience of depth and whether spatial experience qualifies as 2D, 3D, or somewhere in between (see Note 12 in Appendix B). We also did not address how the feeling of a perspectival ‘center’ of space [24] might be specified. Apart from some asymmetries due to border effects (Figure 4), a natural center may be provided by grids specifying body space, which would be heavily bound by relations to the middle of visual space, and by grids responsible for initiating movements near the body midline. We also did not consider how causal distinctions and relations specified by units arranged hierarchically, in an inverted tree-like fashion, can be bound with those specified by grid-like areas, corresponding to the phenomenal binding of objects in space. Nor have we considered the possible role of units arranged into local cliques at every position in grid-like areas in specifying qualities such as color, under the assumption that color, too, can be accounted for in structural terms.

On the other hand, the results presented here suggest further correspondences between properties of phenomenal space and properties of cause-effect structures specified by grids. Thus, just as nearby spots are phenomenally similar with respect to their region, location, and distances within the space, nearby sub-structures are similar with respect to their subtext and supertext. Furthermore, just as the overall structure of experienced space is largely stable regardless of local inhomogeneities such as stars, edges, objects and so on, the cause-effect structure specified by a grid is largely stable regardless of local inhomogeneities triggered by changes in the state of the grid’s units or by increased or decreased salience associated with attentional highlighting. Finally, we have shown that, just like experienced space does not have a boundary, in the sense that there is no further space exterior to it [25], the cause-effect structure specified by a grid also has no exterior and therefore no boundary at its ‘edge’. 

We have also shown that this spatial cause-effect structure, characterized by extendedness, is specified by grid-like substrates (Figure 8A) but not by other kinds of substrates, such as ones with units connected at random (Figure 8B). However, a general characterization of the relationship between the organization of substrates and their unfolded cause-effect structure, and of the features of lattice-like substrates that allow them to specify cause-effect structures of a spatial kind, remains beyond the scope of this paper. 

### 4.4. Introspection: An Indispensable but Imperfect Tool to Bootstrap a Physical Account of Phenomenal Properties

Because the structure of experienced space is at least partly penetrable, we can employ introspection to attend any spot and to characterize the fundamental properties that make it feel extended—through reflexivity, connection, fusion, and inclusion with other spots—as well as derived properties such as the region of space it occupies, its location within the overall canvas, its size, boundary, and distance from other spots. Doing so allows us to assess the proposed correspondence between the phenomenal structure of space and the cause-effect structure specified by a grid. To the extent that the correspondence can be considered as validated, it is reasonable to infer that the fabric of space as phenomenal extension remains largely the same when we are not introspecting and attending specific spots: every spot is ‘there’ where it belongs—at its particular location and distance from every other spot—right in front of our eyes, without any effort on our part. In accordance with this inference, we have shown here that the cause-effect structure specified by a grid is an extension regardless of the presence of inhomogeneities or attentional highlights.

While introspection is an essential tool to validate the proposed correspondence between phenomenal and physical properties, it comes with many limitations. Indeed, once a correspondence is tentatively established, an analysis of the cause-effect structure specified by a substrate can reveal features that are typically not available to introspection. For example, it may be nearly impossible to penetrate through introspection down to the level of the individual distinctions or relations that are assumed to compose all of experience. As illustrated here, attention can be simulated by increasing the excitability of a subset of units, as could be done by back-projections from higher-level to lower-level, topographically organized cortical areas. Doing so, however, highlights not just a single distinction, but a compound distinction and an associated compound context of relations (Figure 13) (see Note 13 in Appendix B). Thus the correspondence between phenomenal and causal properties can only be validated coarsely through introspection—at the level of compound distinctions and contexts. That such a correspondence may hold all the way down to individual distinctions and relations is therefore an inference—albeit an inference from the best explanation.

Another limitation of introspection has to do with our inability to divest our experience of the phenomenal properties under study—in the present case those of spatial experiences—of extraneous phenomenal contents. As was argued in the Introduction, ideally we would like to isolate those properties of experience that make it spatial, abstracting away from non-spatial properties. For example, within visual space, we can reason that the canvas could be white or black, red or green, the inhomogeneities dark over a light background, or the other way around, yet its spatial features remain largely the same, regardless of its local qualities. Similarly, we can try and attend to a spot as a purely spatial distinction, as if we did not have the concept of ‘spot’ in our mind, not to mention the more general concept of ‘visual’. However, it is impossible for us to truly experience space without seeing its color, or without seeing a spot as an instance of the concept ‘spot’. The very richness of distinctions and relations that compose spatial experience suggests that the number of other distinctions and relations that compose an experience, even when merely introspecting empty space, must be even larger, and cannot be prevented from contributing to it, making the isolation of purely spatial properties a non-trivial exercise. 

### 4.5. Some Points of Contact with Mereology and Topology 

It is worth briefly considering how the present approach relates to classic disciplines that study abstract properties of parts and wholes (mereology), abstract properties of space (geometry and topology), or both (mereotopology, [26]), notably in their discrete, atomic version [27]. Because the development of these disciplines is necessarily grounded in our own phenomenology, especially the phenomenology of spatial experience, it is not surprising that there should be many points of contact. For example, the present approach shows that the causal relations between two causal distinctions A and B can be characterized as follows: A’s cause-effect is included (up) by B’s; B’s cause-effect includes (down) A’s; A’s cause-effect includes B’s and B’s cause-effect includes A’s; A’s cause-effect partially overlaps B’s; and A’s cause-effect is not related to B’s. Similarly, a set of five jointly exhaustive and pairwise disjoint relations characterize mereology [27], namely: A is a proper part of B (corresponding to inclusion down as discussed here); B contains A as a proper part (inclusion up); A coincides with B (mutual inclusion); A partially overlaps B (connection); and A is disjoint from B (no relation). There are also points of contact with geometry and topology, and more specifically with discrete mereotopology [27]. For example, extendedness as defined here shares some aspects with the definition of topological spaces and with closure operations. Moreover, notions such as regions, boundaries, distances, and so on, are defined in ways analogous to discrete mereotopology. Finally, a case could be made that fundamental set-theoretic notions such as intersection and union may also find their origin in fundamental properties of spatial experience such as connection and fusion.

Nevertheless, there are also some key differences between the present approach and that of these abstract disciplines. An obvious difference is that here a ‘part’, taken as a sub-structure, is composed of *both* distinctions *and* the relations that bind them in a particular way. Classic extensional mereology, instead, ignores relations. The most important difference, however, is that all the ‘parts’ considered here are required to *exist*: in phenomenal terms, they are required to exist as components of an experience; in physical terms, they are required to exist as sub-structures of a cause-effect structure. As an example, among all possible distinctions and relations, only those having maximally irreducible cause-effect power exist in a causal, intrinsic sense, while those that are reducible do not (Figure 4). In classic extensional mereology, by contrast, all possible parts are on equal footing—a principle called unrestricted composition. With respect to space, the fundamental relational properties of spatial experience considered here—reflexivity and extendedness, comprising connection, fusion, and inclusion—as well as derived properties such as regions, locations, sizes, boundaries, distances, inhomogeneities, and highlights, can be accounted for systematically in terms of intrinsic causal properties of the cause-effect structure specified by a grid, without any abstract, extrinsic ingredient (see Note 14 in Appendix B). By contrast, in topology and mereotopology similar notions are defined abstractly and extrinsically, rather than causally and intrinsically (see Note 15 in Appendix B).

### 4.6. Grids in the Brain as a Substrate for Spatial Experience 

The proposal that cause-effect structures specified by grid-like networks can account for many key features of spatial experiences leads naturally to the hypothesis that the neural substrate of spatial consciousness might be provided by grid-like networks in our brain. Many areas of the cerebral cortex, especially in its posterior regions, are organized topographically, meaning that nearby units within each area show similar response properties with respect to sensory stimuli [28]. Systematic variations of stimulus parameters, such as the position of visual stimuli in external space, typically map ‘topographically’ onto systematic variations of response properties on the cortical surface [29,30,31]. Areas dealing with non-visual modalities, such as touch, are also organized like maps—for example, primary somatosensory cortex maps bodily locations in an orderly way [28]. 

Within each area, units sharing response properties are also preferentially connected. For example, the anatomical connectivity of primary visual cortex is such that neurons that are close to each other are strongly connected, whereas neurons that are far away may not be connected at all [32]. Similar arrangements have been described for areas higher up in the visual hierarchy, as well as for sensory areas in non-visual modalities. The converging feedforward/diverging feedback connectivity between hierarchically organized grid-like cortical areas is also topographically ordered, suggesting that much of posterior cortex may be considered as a stack of grids, where each node has both lateral intra-areal and feedforward/feedback inter-areal connections to neighboring nodes [33,34] (see Note 16 in Appendix B).

Altogether, a rough calculation indicates that topographically organized areas may make up more than one third of the cerebral cortex of humans, and even more in rodents because of the predominance of sensory cortices. It should be emphasized, however, that not all topographically mapped areas are necessarily connected in a grid-like manner; that is, maps are not necessarily grids. Thus, thalamic nuclei that are topographically organized in their responses to sensory stimuli and provide topographically mapped reciprocal connections to cortical areas are not grid-like, as they lack lateral connections among relay neurons [35]. Conversely, grid-like organization and topographic mappings are found in brain regions outside of the thalamocortical system, such as the superior colliculus [36].

Most important for the present purposes, grid-like cortical areas appear to be directly involved in experiencing space. For example, broad unilateral lesions of visual cortical areas produce contralateral blindness of which patients are typically not aware (hemianopia, [37,38]). From their intrinsic perspective, one side of visual space ceases to exist altogether—it is simply ‘not there’. Indeed, after unilateral resection of the occipital lobe, the extent of imagined space shrinks (‘how large is an elephant’, [39]). Split-brain patients, speaking through their left hemisphere, are also unaware of the left side of the visual field [40]. Even an indirect deactivation or disconnection of grid-like visual cortices may lead to the disappearance of one side of space, as is the case in hemineglect [41,42]. Again, patients are typically not aware of lacking a portion of space and they may only realize their deficit indirectly, because they bump into objects or are told by others. In hemineglect, too, the extent of imagined space shrinks [43]. If a smaller portion of posterior cortex is lesioned, patients have scotomas (local blindness) for the corresponding portion of space. In this case, too, the blind region does not look dark or empty, but is simply not seen [38]. This is unlike the case of artificially-induced scotomas that leave visual cortex unchanged and the scotomatous region spatially intact and ‘filled-in’ [44], see also Note 17 in Appendix B).

Stimulation and recording studies provide evidence complementary to lesions studies. Stimulation experiments, whether through intracranial electrodes or through magnetic pulses applied transcranially, show that direct activation of grid-like areas in both primary visual cortex and parietal cortex can produce phosphenes that are localized in experienced space consistent with their topographic organization [45]. Recording studies using sensory stimuli have demonstrated a systematic correspondence between where activations occur in visual cortical areas (such as V1–V2 and V5) and where certain features are experienced in subjective 2D space. This is true not only when we look at a screen, but also when we imagine or dream objects as located at particular positions in visual space [46,47]. Indeed, there are now clinical trials for direct visual cortical stimulation to produce phosphenes in blind patients. In summary, there is a systematic correspondence between positions in the external world, positions in grid-like cortical maps, and locations in experienced space. Paradoxically, it is the very straightforwardness of this correspondence that may conceal the difficulty of accounting for why space feels the way it does (see Note 18 in Appendix B).

### 4.7. Some Tests and Predictions

If phenomenal spaces correspond to cause-effect structures specified by cortical grid-like substrates, then changes in such substrates should lead to predictable changes in spatial experience. An interesting question is whether phenomenal features of certain scotomas, such as filling in, ‘warping’, or ‘sewing’ of the surrounding space, can be predicted from features of the cause-effect structure specified by cortical grids, depending on the underlying cause. Counterintuitive predictions are especially important. For example, in the view presented here, a cortical grid should support the experience of space whether it is active or inactive, as long as it is in working order—that is, an *inactive* neuron is fundamentally different from an *inactivated* neuron, because the former still has causal powers in the system [7]. Thus, the dark sky or a blank screen should still be experienced as spatially extended, even though activity in cortical grids may be at background levels. Background activity in cortical grids should support a pure experience of space also in the absence of perceived objects and while disconnected from external inputs, as can be achieved during certain dreams [48] or meditation states [49]. A related prediction is that changes in the strength of lateral connections in a grid-like area should be associated with changes in spatial experience, even if neural activity did not change [7]. To begin testing this prediction experimentally, we sought to induce local changes in connection strength by repeatedly presenting synchronous point-like stimuli at two cortical positions linked by lateral connections [50]. After training, point-like stimuli presented at untrained positions further apart appeared closer than before. This result is consistent with a contraction of experienced space due to changes in connection strength rather than to changes in neural activity. Other findings potentially consistent with this interpretation have also been reported [51]. Lateral connections in cortical grids such as V1 are usually thought to serve ancillary functions, such as modulating ‘information-processing’ carried out by feed-forward connections [52,53,54]. According to the present proposal, instead, the lateral connections that constitute grid-like networks are responsible for experiencing space *tout court*, whether or not they carry neural activity. Thus, if lateral synaptic connections were inactivated without inactivating the associated pre- or post-synaptic neurons, the experience of space would cease, irrespective of any activity along feedforward connections. A complementary prediction is that, in principle, properly extending a cortical grid with additional neural hardware, such as implanted cortical organoids, should extend experienced space.

The proposal that grid-like areas, primarily located in posterior cortex, specify cause-effect structures that correspond in a one-to-one manner to the experience of space also provides a novel angle on long-standing controversies about the nature of phenomenal space. Some have argued that it has largely Euclidean properties [55], others a spherical geometry [56], others hyperbolic [57], yet others emphasize how its geometrical properties vary with eccentricity, the presence of objects, attention, and even task setting [58,59]. If the structure of experienced space corresponds to the cause-effect structure specified by cortical grids, as proposed here, such complexity would be expected. This is because the precise features of the experience would depend on the precise pattern of connections, which varies with eccentricity and other factors, as well as on changes in connection strength associated with the presence of objects, attentional modulation, and on other idiosyncratic influences, such as early visual experience of individual subjects (see Note 19 in Appendix B).

In principle, this proposal should also lead to testable predictions concerning other features of phenomenal space: the way space extends across the midline thanks to callosal connections; the way higher-level grid-like areas contribute to the experience of space thanks to convergent/divergent, topographically mapped connections to lower-level areas, as well as to the binding of objects to space; the binding of experienced ‘retinotopic’ space with experienced ‘body space’ (where visual space is placed with respect to our body), likely mediated by parietal areas; and with experienced ‘environmental space’ (where the surrounding environment is placed with respect to our body and visual space), possibly mediated by retrosplenial cortex [60].

### 4.8. Can Other Approaches Account for Spatial Experience? 

The one-to-one correspondence proposed here, between properties of spatial experience and properties of the cause-effect structure specified by a grid-like substrate, means that all the phenomenal distinctions and relations that exist in our mind, and the sub-structures they compose, must exist as causal distinctions, relations, and sub-structures specified by grid-like substrates in our brain. Crucially, the correspondence is not with the physical substrate as such—the set of nerve cells one can operationally observe and manipulate—but with the cause-effect structure it specifies: its cause-effect powers, properly unfolded. 

Because space is usually taken for granted, it is not easy to pinpoint explicit alternative accounts with which to compare the present one (see Note 20 in Appendix B). Nevertheless, it may be instructive to consider whether or not the properties of spatial experience could be accounted for by appealing to representations of the environment, behaviors and functional capabilities, neural states, or neural substrates as such.

Consider representations of the environment first. One might envision that the spatial structure of the external world is the referent of spatial experience, hence topographically mapped cortical areas merely need to ‘represent’ the environment, as sampled through stimulus space, to inherit its spatial structure. However, leaving aside the nature of external space itself, it is not clear how the structure of phenomenal space, as experienced from the intrinsic perspective of our conscious mind, would be inherited from something extrinsic to it. This problem is especially obvious for spatial experiences that occur when we are disconnected from the environment, as when dreaming of the starry sky. If the experience feels spatial, it must feel so because it actually has spatial properties intrinsically, when it is dreamt, and not by inheriting spatial properties from an external environment to which the brain was exposed in the past. Of course, in the course of evolution, development, and learning, causal properties of the environment do mold the neural substrate of spatial experience (see Note 21 in Appendix B), but at issue is what corresponds to the structure of experience here and now, not how it came about. It should also be clear that stimuli from the environment are not spatially organized in themselves: there is no extendedness, no region or location, no size, boundary or distance in a sensory stimulus unless one presupposes that space already exists intrinsically in the mind of an observer (see Note 22 in Appendix B). 

If an appeal to a representation of the environment tries to shift the burden to the input side—to upstream causes—an appeal to spatial behaviors and functions tries to shift it to the output side—to downstream effects. We can obviously report where something is, whether a larger spot includes a smaller spot or not, whether two stars are close or far, and so on. In functional terms, if one can account for all our spatial behaviors and functions by a series of ‘processing’ steps along topographically mapped regions of our brain, then nothing else would seem to need explaining. Such a functionalist approach, if it does not outright deny the reality of one’s experience, at best treats it as an ‘illusion’ [61]. Even so, it certainly fails to provide a one-to-one account for the structural features that make the ‘illusion’ feel the particular way it does—in the present case, that it feels extended. In other words, it has nothing to say about the countless distinctions and relations that compose the experience of extendedness as it exists phenomenally, and focuses exclusively on the few functions that can be performed one at a time. Indeed, the dissociation between the richness of an experience—say, experiencing the starry sky or even a blank canvas—and the poverty of behavioral reports—say, reporting the position of a stimulus in the visual field—can lead to a paradoxical denial of the primary evidence of our own consciousness: rather than admit that the richness of what we experience cannot be explained by the poverty of what we do, some scientists insist that we cannot possibly experience so much given that we can report so little [62]. Note also that, in strictly functional terms, there would be no fundamental difference between an embodied computer program simulating all our spatial behaviors and functions (functional equivalence) and the humans it is simulating, who actually experience space and don’t just behave as if they did (no phenomenal equivalence, Findlay, Marshall, et al., unpublished).

Alternatively, one could maintain that properties of experienced space are properties of certain neural states, irrespective of what those states may represent or of what functional consequences they might have. And indeed, it is possible to ‘decode’ spatial properties from patterns of neural activity in topographically organized cortical areas, whether stimuli are experienced left or right, close or far, and so on, with increasing reliability and precision [63]. However, leaving aside that one can decode spatial properties just as well from the retina and, for that matter, from the sensor of a digital camera, a neural state by itself cannot provide a one-to-one correspondence—an isomorphism—with the distinctions and relations that compose extended spots, regions, locations, sizes, boundaries, distances, and so on (see Note 23 in Appendix B). When we decode and interpret a neural activity pattern in a topographically mapped cortical area as spatially meaningful, it is because we presuppose a spatial organization, but that organization is already in our mind—the mind of the beholder—not in the activity pattern as such (see Note 24 in Appendix B). Without a causal context, a neural state is just as meaningless as an array of 0s and 1s in a computer chip (see Note 25 in Appendix B).

Finally, one could propose that the properties of experienced space might be associated not with a neural state, but with a neural substrate—not just a pattern of neural activity, but also the underlying connectivity as it evolved by exposure to the environment. For example, the multiscale receptive field organization along the cortical hierarchy may seem well-suited to implementing a partial ordering [64,65]. Thus, if two neural units are neighbors in a grid-like network, their neighborhood could be ‘encoded’ by a unit higher-up in the visual hierarchy to which their outputs converge, and so on recursively across multiple scales. From a functional perspective, the set of multiscale receptive fields orders the units in the first level such that output units at higher levels of the hierarchy encode contiguous regions of visual space, and their own outputs can be combined to guide spatial behaviors. Along similar lines, one could propose that the lateral connections among units in grid-like areas might ‘encode’ neighborhood relations. Thus, if two stars look close together, one would find that their point images in the cortex are separated by few synaptic steps, but by many synaptic steps if they look far apart. However, neither the hierarchical organization of receptive fields, nor a grid-like connectivity as such, can provide a one-to-one correspondence with the structure of spatial experience. In the case of the hierarchy, for instance, the fact that unit A at the lowest level is 2 units away from C and 6 units away from G is not directly ‘encoded’ by any unit at the next level up. Similarly, there are no neural units that could ‘encode’ every distance relation present in phenomenology (see Note 26 in Appendix B). The same applies to inclusion relations corresponding to phenomenal regions and locations (see Note 27 in Appendix B).

### 4.9. The Extraordinary Richness of Space and Its Unfolded Physical Correspondent

In this paper, the proposal that the properties of spatial phenomenology correspond one-to-one to the properties of cause-effect structures specified by grid-like cortical substrates could only be formulated in a bare-bones, rudimentary manner (see Note 28 in Appendix B). Nevertheless, if this account is pointing in the right direction, it should make us reconsider the extraordinary richness of both phenomenal experience and its physical correspondent. We often take space for granted because it seems easy to describe: what could be simpler than a blank, empty canvas extending in all directions? But as we have tried to illustrate, to experience empty space as such requires the existence of a multitude of distinctions and relations, composing a multitude of extended spots, regions, locations, and distances (see Note 29 in Appendix B). Cortical grids, the proposed substrate of spatial experiences, also seem remarkably easy to describe—a 2D array of units linked by near-neighbor connections. But when a simple grid is unfolded to reveal its cause-effect powers—all the causal distinctions and relations it supports—the richness of the cause-effect structure it specifies boggles the mind (see Note 30 in Appendix B). And so it should be, because the richness of spatial experience should be matched one-to-one by the richness of its physical correspondent (see Note 31 in Appendix B). Usually, we don’t stop in amazement at the sheer numerosity of the phenomenal distinctions and relations that exist, ‘right in front of our eyes’, when we look at the night sky. Perhaps, on occasion, we should.

## Appendix B

1. The term *space* can be taken to refer to a variety of concepts: there is the *physical space* of the world that we take to exist independent of our knowledge of it (“outer space”); there is *spatial cognition*, the psychological model of physical space that an animal builds and uses to navigate and understand the world; and there is *phenomenal space*, the fact that some aspects of experience have a spatial structure. Obviously these three types of space are interlinked in various ways (spatial cognition is usually “about” some physical space, and spatial experience is often involved in spatial cognition), but they are fundamentally distinct. The focus of this paper is exclusively on phenomenal space. Partly due to its pervasiveness, space also underlies metaphors at various levels of abstraction in both thought and language [66].
2. In other words, it is always the case that “the blot is on a spot”.
3. In previous work distinctions were called concepts (forming a conceptual structure), but the term has been abandoned because many emphasize the separation between perceptual and conceptual aspects of experience. More significant changes to the formalism of integrated information theory detailed in this paper include the replacement of the Earth Mover’s Distance (EMD; Oizumi et al 2014) with the ‘intrinsic difference’ (Barbosa et al. unpublished) as the means of identifying a mechanism’s maximally irreducible cause and effect (that is, of computing the *φ* value of a distinction or relation), and the introduction of a method for determining relations between distinctions. The EMD was originally used because its assumption of a ‘state space’ made relations between distinctions (concepts) implicit in the structure of the causes and effects. By making the relations explicit, the original rationale for the EMD is obviated. The intrinsic difference measure is derived from the postulates of integrated information theory and is proven to do so uniquely; Barbosa et al).
4. Relations are essential not just for binding distinctions with each other, but also for establishing the identity of units and unit states. From the intrinsic perspective of the system, the units in a mechanism’s purview as well as their states do not come with an extrinsic ‘label’ that identifies them (such as unit A or B, state 0 or 1). It is relations that specify which units in a mechanism’s purview correspond to which unit in the purview of other mechanisms, and which states correspond to which states.
5. The simplicial complex description is apt because the possible existence of higher-order relations is constrained by the existence of lower-order relations, and this is an essential part of the definition of the simplicial complex. That is, just as a triangle cannot exist if one of its edges does not exist, there cannot exist a relation over S if there is not a relation over every (non-empty, non-singleton) subset of S. However, the presence of a set of lower-order relations does not dictate existence of higher-order relations (there may be three 2-relations over three causes or effects, and yet no 3-relation).
6. In the case of vision, we have learned that external space is ‘mapped’ first onto the retina, then onto certain thalamic nuclei, and then at different levels in the central nervous system through a series of ‘retinotopic’ maps, with many complex transformations in between. At some point there must occur a transformation from ‘retinotopic coordinates’ to ‘spatiotopic coordinates’, which allow us to grab a cup at its actual position in external space independent of its position on our retina. Further on, the brain constructs high-level ‘representations’ of the environment, mediated among others by place cells in the hippocampus, grid cells in entorhinal cortex, and by cells in retrosplenial cortex. Other questions of interest concern how the brain adds depth to 2D space, and how representations of space in different modalities are kept in register.
7. With exceptions such as Kant [55], Lotze [5], James [4], Mach [67], Smythies [68], Gur [69], and Koenderink [70]. For example, Lotze argued that the location of a point in space was just as qualitative as its color, hence nerve fibers must carry local signs for both (though how the mind would “apprehend” either was not explained). James argued that the feeling of extendedness must be a primitive quale, like that of pain. And just like pain grows more painful, extendedness would grow larger with a stronger excitation of nerve fibers. More often, even phenomenological studies involve a mixture of notions of internal and external space, as in Wagner [59] and Masrour [71,72].
8. According to integrated information theory, colors must correspond to causal sub-structures. The reason they may be difficult to introspect could be because, unlike in the case of space, we lack the ability to deploy endogenous attention through precise back-connections to transiently modify the underlying circuits and thereby affect how colors feel. However, we could certainly modify the neural substrate of color exogenously through precise electrical stimulation or the application of drugs, thereby changing the cause-effect sub-structure in a way that should lead to systematic, predictable changes in how color is experienced.
9. In integrated information theory, this one-to-one correspondence is meant as an explanatory identity: an attempt to account for the properties of experience—whose existence is given immediately and indubitably—in terms of properties of cause-effect structures, which can be established operationally, through observations and manipulations of a physical world postulated from within consciousness. As an identity, it should not be considered as an isomorphism between two different ‘things’.
10. It is interesting to consider to what extent this can be true distance metric, being 0 between a point distinction and itself, symmetric, and satisfying the triangle inequality. As was shown here by simulating the effects of attention on compound distinctions and relations (contexts), these inherit extendedness and derived properties from the extendedness of the distinctions that compose them.
11. Furthermore, if only the top-order purview were missing, one would still experience the extendedness of smaller spots, but lack the feeling that they are part of a single, larger space—a kind of ‘agnosia’ for space as a whole. Conversely, if only the bottom-order purviews were missing, one would still experience space, but in a less detailed manner.
12. The dimensionality of space can be characterized in multiple ways, see for example Poincare [73], French [74], Lebesgue [75]. Some have argued that perceived depth may be considered as a local quality of 2D space, not unlike color [74,76]. A possibility suggested by the present analysis is that the cause-effect structures specified by 1D and 2D grids can be characterized as differing in the minimum order of *connection* (2 or 3, respectively) that is available for extending a lower-order sub-structure (see Appendix C).
13. This scenario implies that the classic structuralist hope of isolating the ‘atoms’ of experience through introspection (as in Külpe and Titchener, [77]) is likely to be ill-posed. However, as was mentioned in footnote 8 about color, even if we cannot manipulate the substrate of experience with sufficient precision endogenously through introspection, it is in principle possible to manipulate it exogenously though electrical stimulation or other means. More generally, the proposed correspondence between phenomenal and causal structure can be tested far beyond what is achievable directly by introspection.
14. Alternative but equivalent characterization of extension (connection, fusion, inclusion) may be possible, just as there are equivalent axiomatic characterizations of mereology and topology.
15. Topology and mereotopology provide additional criteria such as connectedness (reminiscent of the requirement for irreducibility) and compactness (reminiscent of the requirement for definiteness).
16. Many other notable features of connectivity within grid-like areas should be considered, such as the higher density and specificity in supragranular layers [78,79], the organization of modules into hexagonal lattices [80], and the further specialization of units within each topographic module.
17. A few patients with lesions restricted to a portion of early visual areas have blindsight: they can still detect simple features of visual stimuli above chance, but deny seeing them consciously, at least in the sense of seeing something located in space within the blind field. Intriguingly, patients may report having feelings, for example of movement, possibly due to activation of higher-level areas [81]. However, such feelings are not experienced as bound to an object located in visual space. Other patients with lesions restricted to a portion of early visual areas (primarily V1) still report some spatial experience, occasionally occupied by hallucinations [82]; [83], perhaps because some grid-like areas beyond V1 may still be functional. See also [84].
18. It is less clear to what extent V1 and other primary grid-like areas, as opposed to higher level grid-like areas, contribute to experience [85]. With a few possible exceptions [86], most of the negative evidence concerns the contribution of primary areas to local qualities of experience, such as line orientation, rather than their contribution to spatial experience as such [87].
19. It may be especially important to consider the effects of the activation of NMDA receptors, of short-term synaptic plasticity, and of other activity-dependent changes in connection efficacy, as well as that of inhibitory interneurons. Such effects may markedly change the cause-effect structure specified by the same anatomical grid, possibly even expanding or restricting the extent of the substrate having maximal cause-effect power.
20. A rare exception is a ‘TV raster’ mechanism suggested by Smythies [68].
21. In this sense, the cause-effect structures specified by neural grids that map visual or somatosensory input should match the causal structure of the environment itself (for example, its ‘smoothness’).
22. As already observed by Lotze, the arrangement of fibers within the optic nerve is not intrinsically spatial and could be scrambled without any effect on experienced space; after all, the arrangement of nerve fibers within the olfactory nerve has nothing to do with space.
23. Moreover, a neural state as such cannot even satisfy the essential requirements of any experience, such as intrinsic cause-effect power, composition, irreducibility, and so on [20]. It is also important to realize that any attempt to attribute a “local sign” to the state of some neurons encounters the problem of how to attribute to that state its particular meaning. Consider what might happen in the brain when one is experiencing a bright blot in the upper right quadrant of an otherwise dark canvas, say while doing absolutely nothing (no task, no report), or even better while dreaming. Based on neurophysiological studies, one would expect that the neural correlate of such an experience should correspond to a patch of highly active neurons at appropriate topographic positions in various areas in the visual cortical hierarchy, surrounded by inactive or minimally active neurons at all other positions. In fact, for the purpose of the example, the inactive neurons around the active patch might as well be inactivated or their connections blocked. How could a patch of active neurons, by themselves, convey the meaning (and feeling) of a region of inhomogeneity at that location, its boundary and distance from all other spots? In other words, how could their firing signal that there is a bright spot ‘here’, without all the ‘there’ and their relations to the ‘here’? After all, cortical neurons do not come with a label that conveys their meaning—in this case, the meaning of ‘here’, not to mention that no other neurons would be able to understand it. Of course, one might then fall back onto representational or functionalist views, which try to avoid the impasse by redirecting the meaning either to the input, and eventually the environment, or to the output, and eventually a behavior, as discussed above.
24. Neurophysiologists sometimes talk of explicit representation if a variable of interest is represented by an activity pattern over a set of neural units that can be ‘read out’ by downstream neural units using a neurobiologically plausible mechanism, such as linear or radial-basis-function decoding [87,88,89,90]. When looking at a face, a pattern of activity over the retina does not represent the face explicitly, because extracting the variable of interest from it requires a sequence of non-linear transformations; by contrast, patterns of activity higher up in the visual hierarchy represent a face explicitly enough that they can be employed for image categorization without requiring much processing. However, as was argued above, patterns of activity in either low- or high-level areas cannot possibly represent one-to-one the relations that compose extended spots, regions, locations, sizes, boundaries, and distances.
25. Additional features, such as synchrony of neuronal firing, do not offer much help. First, the relational structure of space is present in full even when we experience a perfectly blank canvas, which is likely associated with minimal activity in grid-like areas. Moreover, synchrony as such cannot provide the substrate of relatedness: if it were so, strictly speaking all neurons that happen to fire together, at whatever time scale one deems relevant, should support related components of experience, not only within the same brain, thus extending to the cerebellum, the spinal cord, and perhaps the enteric nervous system, but even across multiple brains. If additional criteria are considered—say, the fact that synchronously firing neurons can have stronger effects on some joint target—which would then read out the synchrony—then it is not synchrony as such that matters, but its effects downstream, as discussed below. Gestalt psychologists classically proposed an isomorphism between extended forms in experience and electric fields generated by neural activity to be found at an appropriate level in the brain [91,92]. However, the extendedness of spatial experience was not explained, but merely inherited through isomorphism with a physical correspondent—the field—that was assumed to be spatial by being extended in physical space.
26. An intriguing finding of recent fMRI investigations in humans is that dozens of areas (mostly in posterior cortex) mapping positions in visual stimulus space do so retinotopically, in line with the retinal position of an object, rather than spatiotopically, in line with the position of an object in external space, independent of eye position [29]. Thus, spatiotopic object position, which must be calculated to properly move one’s hand to grasp an object based on its retinal position while discounting eye position, seems to be recomputed “on-demand” with each eye movement. Intriguingly, these computations remain unconscious. Golomb and Kanwisher argue that our phenomenology is spatiotopic, rather than retinotopic (or V1-V2-topic), but they confound the phenomenal impression of stability of the external world when we move our eyes for the actual experience of phenomenal space: when we move our eyes to the left, the visual field does extend to the left and restrict on the right, even though no movement is perceived.
27. It is also useful to consider how one could represent this information in a computer, rather than in a physical grid-like network. For example, a computer may encode a value, say 0 or 1, for the full range of X and Y Cartesian coordinates. In the case of a blank screen it might encode 0 for all pairs of X and Y. Or it might encode some compressed representations, which in this case could be much shorter: 0 at all coordinate pairs. It could of course encode the position of two bright dots as a value of 1 at the appropriate X and Y pairs. On demand, it could then easily compute the distance between them. Of course, on demand it could also compute the distance between any bright dot and any other position on the screen, that is, any other pair of Cartesian coordinates. Furthermore, it could compute extended ‘spots’ as containing a region, in the sense of a subset of pairs of Cartesian coordinates, and do the same for their location. And of course, it could readily recompute any desired result every time the image on the screen changes. Once again, however, this structured information is not immediately available but requires an immense number of on-demand computations.
28. Unfolding the cause-effect structures specified by actual cortical areas, taking into account the relevant units at the relevant grain—neurons or minicolums, tens or hundreds of milliseconds [20]—and their detailed connectivity is currently unrealistic.
29. Said otherwise, experiencing space means to be ‘presented’ with an immense amount of structured information (in the integrated information theory sense).
30. Here we are not considering physical constraints on the mechanisms and connections of grid-like substrates that ultimately determine how large a cause-effect structure they can specify. In general, the actual size of the maximum of irreducible cause-effect power will be limited by the degree of indeterminism that is intrinsic to each mechanism or provided by uncontrolled external factors (Barbosa et al., unpublished).
31. The so called “binding problem” is usually brought up to underline the need for the brain to have mechanisms, such as synchrony of firing, that can “bind”, say, an object to its color. But once one considers the nature of experience—any experience—the binding problem turns out to be the problem of relations—ubiquitous and immense in scope and number: it begins in a major way with space itself, bringing together extended spots into a single, extended canvas.

## Appendix C

When discussing spatial cause-effect structures, a natural question regards their dimensionality: is there a sense in which a phenomenal space has identifiable dimension? The covering dimension of Lebesgue is the most basic topological definition of the dimension of a space, and here we show how it may be applied to extended cause-effect structures. Below is the definition of Lebesgue’s covering dimension from Wikipedia (The same definition, in more technical language and distributed over several sections, is found in [75])

“An *open cover* of a topological space *X* is a family of *open sets* whose union contains *X*. The *ply* or *order* of a cover is the smallest number *n* (if it exists) such that each point of the space belongs to at most *n* sets in the cover. A *refinement* of a cover *C* is another cover, each of whose sets is a subset of a set in *C*; its ply may be smaller than, or possibly larger than, the ply of *C*. The covering dimension of a topological space *X* is defined to be the minimum value of *n*, such that every open cover *C* of *X* has an open refinement with ply n + 1 or below.”

The Lebesgue covering dimension is a way of defining the dimension of a *topological* space. While the cause-effect structure of a grid is not a topological space (for example, the grid does not specify anything like the empty set), it is not difficult to formulate a *Lebesguesque* covering dimension for an extended cause-effect structure.

Using the above definition of the covering dimension, we define an analogous covering dimension based entirely in components of the cause-effect structure:

(i) A *topological point* is analogous to a “point distinction”, which is ordered by extended distinctions but is not itself extended. For the 8-node grid structure, point distinctions include those with one-unit purviews over A, B, etc.
(ii) An *open set* is analogous to any distinction that is extended both *up* and *down*. Any such distinction can be (recursively) decomposed down to a set of purely up-extended distinctions called its *basis*. For example, the extended distinction $\frac{CDEF}{\langle CDEF^{i},CDEF^{o}\rangle }$ can be decomposed to the basis $\{\frac{CD}{\langle CD^{i},CD^{o}\rangle },\frac{DE}{\langle DE^{i},DE^{o}\rangle },\frac{EF}{\langle EF^{i},EF^{o}\rangle }\}\;$.
(iii) A *cover* of an extended distinction is a group of extended distinctions which together include exactly the basis of the covered distinction (the union of their bases are equivalent to the basis of the covered distinction). For example, $\frac{CDEF}{\langle CDEF^{i},CDEF^{o}\rangle }$ has the basis $\{\frac{CD}{\langle CD^{i},CD^{o}\rangle },\frac{DE}{\langle DE^{i},DE^{o}\rangle },\frac{EF}{\langle EF^{i},EF^{o}\rangle }\}$. The distinctions $\frac{CDE}{\langle CDE^{i},CDE^{o}\rangle }$ and $\frac{DEF}{\langle DEF^{i},DEF^{o}\rangle }$ together include the entire basis of *CDEF* and nothing further, so *CDE* and *DEF* are a cover of *CDEF*, and we can refer to these as *covering distinctions*.
(iv) The *order* of a cover of a distinction is the smallest number *m* such that each point distinction included by the covered distinction is overlapped by at most *m* covering distinctions. In the example of (iii), the order of the cover [*CDE*, *DEF*] is *m* = 2, since point *D* (or *E*) is included by two covering distinctions.
(v) A *refinement* of a cover C of some distinction is a set of covers of the distinctions in C. If the distinction *BCDEFG* is covered by C = [*BCD*, *DEFG*], then [*BC*, *CD*] and [*DE*, *EFG*], together, constitute a refinement of C. The order of a refinement is assessed the same way as the order of a cover: the order of this refinement is *m =* 2, since *D* (or *E*) is included in two of the refinement distinctions (*D* is in {*CD, DEF*} and *E* is in {*DEF, EFG*}). The order of a cover is equivalent to the smallest *connection order* that must relate distinctions in the refinement, as shown in Appendix A Figure A1a.
(vi) The covering dimension of an extended distinction (precisely, of a structure of extended distinctions included by an extensive distinction), then, is the minimum value of *l*, such that every cover of that distinction has a refinement with order *m* = *l* + 1 or below.

The measure can be applied to the structure as a whole by considering the covering dimension of the ‘top’ order distinction *ABCDEFGH*. An ideal ‘line grid’ structure (with distinctions {*A, B, C, AB, BC, ABC*, *etc*} would have a covering dimension of *l* = 1 by this measure. The actual cause-effect structure specified by the line-grid is more complicated than this: there are basis distinctions over purviews like BD or CE, so the line grid structure’s dimension is, strictly, *l* = 2 (it’s ‘shape’ is something like a long, flat ribbon).

The same measure can be applied to the cause-effect structure specified by a ‘two-dimensional’ hex grid. As with the line grid, the ‘point distinctions’ of a hex-grid structure will be 1^st^-order distinctions, and the bases all consist of 2-nd-order distinctions. An ideal ‘hex grid’ structure would have a covering dimension of *l* = 2, while an actual hex-grid structure is likely to have dimension *l* = 3 – a planar structure with some ‘thickness’.
